# UPLC–Q–TOF–MS/MS Analysis of Phenolic Compounds from the Fruit of *Cephalostachyum fuchsianum* Gamble and Their Antioxidant and Cytoprotective Activities

**DOI:** 10.3390/molecules27123767

**Published:** 2022-06-11

**Authors:** Yan Wang, Yongqiang Li, Bi Chen, Xianfeng Deng, Qin Luo, Xingru Zao

**Affiliations:** 1 College of Food Science and Technology, Yunnan Agricultural University, Kunming 650201, China; wangyan9612@163.com (Y.W.); dengxf0525@163.com (X.D.); luoqin1526@163.com (Q.L.); zxr1514854119@163.com (X.Z.); 2 School of Life and Health Science, Kaili University, Guizhou 556011, China; 18487194930@163.com

**Keywords:** *Cephalostachyum fuchsianum* Gamble, polyphenol, UPLC–Q–TOF–MS/MS, HepG2 cells, antioxidant activity

## Abstract

Bamboo is a widely distributed graminaceous plant in China and is a potential source of bioactive substances. Incidentally, bamboo’s fruit is rich in phytochemicals such as polyphenols and flavonoids, which are significant to human health. In this study, we identified the phenolic compounds of the fruit and investigated the antioxidant activities of *Cephalostachyum fuchsianum* Gamble (CFG) fruit polyphenols with in vitro and in vivo tests for the first time. UPLC–Q–TOF–MS/MS analysis results showed that the fruit contained 43 phenolic compounds, including 7 hydroxybenzoic acids, 12 flavonoids, 7 coumarins, 10 hydroxycinnamic acids, 1 terpenoid, and 5 lignans. The TPC of SP extracts was higher than that of IBPs extracts in FP and FF. The SP extracts in FP showed better antioxidant activities in vitro compared to those in FF. In addition, polyphenols from CFG fruits protected against H_2_O_2_-induced oxidative damage in HepG2 cells, and the protective effect of polyphenols in FP was superior to that in FF. The analysis results showed that CFG fruit has great potential in exploiting natural chemical substances, which can provide valuable pieces of information for the further development and utilization of CFG.

## 1. Introduction

Bamboo is a perennial one-time flowering plant and is widely known for its economic value and environmental benefits. In addition, bamboo has been used for centuries to treat diverse maladies, including cough, fever, and leprosy [1].

*Cephalostachyum fuchsianum* Gamble (CFG) was named by Gamble J. S. in 1896 [2]. CFG, a member of Bambusoideae (*Cephalostachyum*), is distributed naturally in west and southwest China, Bhutan, Northeast India, and Myanmar. However, similar to other perennial flowering bamboo species, CFG undergoes a vegetative phase for decades [3], followed by massive flowering, bearing fruit, and subsequent death [4]. It is reported that the flowering cycles of CFG generally last for 48 years under natural conditions. Due to their high nutrition and nutraceutical values, CFG fruits have been traditionally used for food and foraging. To date, the fruits of CFG are frequently consumed by local residents as healthy food ingredients, which are stewed in soup with meat, eaten as congee, or consumed like rice [5].

However, to the best of our knowledge, there are few studies on the phytochemicals related to the biological activities of this underutilized fruit. In addition, no one has investigated the cytoprotective activities and antioxidant effects of phenolic extracts from CFG. In order to contribute to facilitating a more comprehensive assessment of the chemical composition in CFG, we identified its polyphenolic metabolites extracts from the fruit flesh (FF) and fruit pulp (FP).

Oxidative stress can lead to the excessive accumulation of reactive oxygen species (ROS) owing to an imbalance between the production of ROS and antioxidant responses. Therefore, bioactive compounds, which have antioxidant properties and the potential to attenuate oxidative stress, are required to prevent or remove oxidative damage. Recently, more attention has been focused on the search for natural phytochemical compounds which might be capable of protecting cells from oxidative damage. Polyphenols are secondary plant metabolites and naturally occurring phytochemicals which display vigorous antioxidant activity. Phenolic compounds are known to reduce oxidative stress and prevent several diseases, including cancer, coronary heart disease, and some cardiovascular disorders [6].

ROS are oxygen-containing reactive molecules, including free radicals such as hydroxyl radicals and superoxide and non-radical molecules such as hydrogen peroxide [7]. Increased production of ROS and reduced innate antioxidant capacity can lead to oxidative stress [8]. The ROS content is detected by measuring fluorescence intensity with a fluorescent probe DCFH–DA. To protect the cells against the detrimental potential of ROS, the body is supported by a defense system that includes both enzymatic and non-enzymatic antioxidants. Generally, the enzymatic systems include the superoxide dismutase (SOD), catalase (CAT), glutathione peroxidase (GPx), and glutathione reductase, while major non-enzymatic systems consist of glutathione (GSH) and ascorbic acid (AsA) and carotenoids, and other dietary antioxidants that scavenge free radicals, leading to the maintenance of cellular redox balance. In view of this, it is necessary to investigate the antioxidant activities of CFG to fill this research gap.

Therefore, the aims of this study were to identify the polyphenolic compounds in CFG fruit extracts and to explore the protective effects of CFG on H_2_O_2_-induced oxidative damage in HepG2 cells.

## 2. Materials and Methods

### 2.1. Chemical Reagent

Methanol, Fetal Bovine Serum (FBS), dimethyl sulfoxide (DMSO), acetonitrile, 3-(4, 5-dimethylthiazol-2-y1)-2,5-diphenyltetrazolium bromide (MTT), acetic acid and 2′,7′-dichlorfluorescein diacetate (DCFH-DA) for liquid chromatography were purchased from Sigma-Aldrich, USA. Folin-Ciocalteu reagent, 6-hydroxy-2,5,7,8-tetramethylchroman-2-carboxylic acid (Trolox), 2,2-diphenyl-1-picrylhydrazyl (DPPH), and 2,4,6-tripyridyl-s-triazine (TPTZ) were obtained from Sigma-Aldrich (St. Louis, MO, USA). 2,2′-azinobis (3-ethylbenzothiazoline-6-sulfonic acid) (ABTS) and H_2_O_2_ were purchased from Aladdin Industrial Corporation (Shanghai, China). HepG2 cells were bought from the Chinese Academy of Sciences cell bank. Dulbecco’s modified Eagle’s medium (DMEM) was purchased from Gibco Life Technologies (Grand Island, NY, USA). In addition, PBS was purchased from Hyclone (Logan, UT, USA). Sodium carbonate (Na_2_CO_3_), sodium nitrite (NaNO_2_), hydrochloric acid (HCL), aluminum chloride (AlCl_3_), and sodium hydroxide (NaOH) were obtained from Sangon Biotech (Shanghai, China).

### 2.2. Plant Materials

*Cephalostachyum fuchsianum* Gamble was purchased from Yingjiang County, Dehong Autonomous Prefecture, Yunnan Province, China, in 2018. The fruits were sealed and placed at −20 °C in a refrigerator at the College of Food Science and Technology, Yunnan Agricultural University, China.

### 2.3. Extraction of Soluble Phenolics (SPs) and Insoluble-Bound Phenolics (IBPs) in CFG

The SPs and IBPs were prepared as in the previously described method [9] with some modifications. The different parts of CFG, including fruit pulp (FP) and fruit flesh (FF) were separated and dried, then ground into power (40-mesh) using a Wiley Mill (Thomas Scientific, Model 4, Swedesboro, NJ, USA).

To extract SPs, 20 mL of 80% methanol was added to 2.0 g of the milled samples, and extraction was performed in an ultrasonic bath (Kunshan Ultrasonic Instrument Co., Ltd., Kunshan, China) for an hour. This procedure was repeated twice, and the supernatants were collected. For the extraction of IBPs, 40 mL NaOH (4 mol/L) was added into the residue after extraction of SPs and placed for 4 h under nitrogen gas conditions. The resultant hydrolysates were acidified to pH 2 with 6 mol/L of HCl, and then centrifuged at 8000× *g* for 10 min at 4 °C (TGL20M, Hunan Xiang Li Scientific Instrument Co., Ltd., Changsha, China). Then, the supernatants were combined and extracted three times with an equal volume of diethyl ether and ethyl acetate (1:1, *v*/*v*). The solvent was evaporated under reduced pressure at 35 °C using a laboratory rotary evaporator.

### 2.4. Determination of Total Phenolic Content (TPC) and Total Flavonoid Content (TFC)

The total phenolic content (TPC) was determined by the Folin-Ciocalteu colorimetric method, as described by Zhu [10] and Li [9]. Briefly, an appropriate amount of the sample was placed in a clean centrifugal tube, then 0.5 mL of 2 mol/L Folin-Ciocalteu reagent was added and mixed. Finally, 1.5 mL of Na_2_CO_3_ solution was added and the volume was fixed to 10 mL with distilled water. After 35 min of incubation in the dark, the absorbance was determined at 725 nm. The TPC in samples was expressed as μmol of ferulic acid (FA) per gram of the dry sample (DS) (μmol FAE/g DS).

The TFC was measured using the aluminum chloride colorimetric method described by Li [9]. Catechin was used as the reference standard, and the results were expressed as μmol of catechin equivalent (CE) per gram of DS (μmol CE/g DS).

### 2.5. Identification of Phenolic Compounds

#### UPLC–Q–TOF–MS/MS Analysis Conditions

Samples were analyzed on an UPLC system (Agilent 1290) coupled to a Q–TOF mass spectrometer (Agilent 6545, Agilent Technologies, Santa Clara, CA, USA). Compounds were separated over a ZORBAX Eclipse Plus C18 column (2.1 × 100 mm, 1.8 µm) maintained at 40 °C. Aqueous 0.05% acetic acid solution (mobile phase A) and acetonitrile (mobile phase B) were used as mobile phase. Gradient elution optimization was performed as follows: 0 min, 5% B; 2 min, 5% B; 10 min, 30% B; 20 min, 100% B. The flow rate was 0.3 mL/min, the injection volume was 2 μL, and the column temperature was maintained at 40 °C. The conditions for negative electrospray ionization mode were as follows: capillary voltages, 3500 V; nebulizer pressure, 35 psi; nozzle voltage, 1000 V; drying gas flow rate, 6 L/min, drying gas temperature, 300 °C; sheath gas temperature, 350 °C; and sheath gas flow, 11 L/min. Full-scan MS was acquired from *m*/*z* 100 to 1000. The isolation window was selected as Medium (*m*/*z*~4) and the collision energy range was 0–30 ev.

The raw mass spectrometry data were processed with the Masshunter Workstation Software: (Agilent Masshunter Qualitative Analysis B.07.00 Masshunter PCDL Manager B.07.00 and Agilent Masshunter Molecular Structure Correlator).

Phenolic compounds were tentatively identified by comparing the MS/MS spectra with the literature, TCM Database @Taiwan (http://tcm.cmu.edu.tw/ (accessed on 22 January 2022)), Metlin (https://metlin.scripps.edu (accessed on 2 November 2021)), and PubChem (https://pubchem.ncbi.nlm.nih.gov (accessed on 23 February 2022)).

### 2.6. Antioxidant Activity Analysis

#### 2.6.1. DPPH Radical Scavenging Assay (DRSA)

DRSA was determined according to Hatano [11] and Yeo [12] with slight modifications. Briefly, a solution of 4 mL 79 μmol/L DPPH in methanol was mixed with 1 mL of the phenolic extract. Then, the mixture was kept in the dark for 10 min at room temperature, and the absorbance was measured at 517 nm. DRSA level was calculated from the calibration curve for Trolox, and the percentage of DPPH radical scavenging (DPPH%) was calculated with Equation (1).

(1) $$\mathrm{DPPH}\;\mathrm{radical}\;\mathrm{scavenging}\;\left(\%\right)=[\left(A_{0}-A_{1})/A_{0}\right]\times 100$$

Here, *A*_0_ is the absorbance of the DPPH solution and *A*_1_ is the absorbance of the sample. The results were expressed as μmol of Trolox equivalent (TE) per g of dry sample (DS) (μmol TE/g DS).

#### 2.6.2. Trolox Equivalent Antioxidant Capacity (TEAC)

The TEAC assay was performed according to the procedure of Li [9] and Zhu [10], with minor modifications. ABTS solution was made by mixing 7 mmol/L ABTS working solution and potassium persulfate (2.45 mmol/L) in a volume ratio of 1:1. The sample solution (100 μL) was mixed with 3.8 mL of ABTS working solution. Then, the absorbance of the samples was read at 734 nm. TEAC level was calculated from the calibration curve for Trolox, and the percentage of ABTS radical scavenging was calculated based on the following equation:

(2) $$\;\mathrm{ABTS}\;\mathrm{scavenging}\;\left(\%\right)=\left[\left(A_{0}-A_{1}\right)/A_{0}\right]\times 100$$

where *A*_1_ is absorbance of the sample and *A*_0_ is the absorbance of the control sample (ABTS solution). The results were expressed as μmol of TE per g of DS (μmol TE/g DS).

#### 2.6.3. Ferric Reducing Antioxidant Power (FRAP) Assay

The FRAP assay of the CFG extract was estimated according to Li’s [9] and Zhu’s [10] procedures. Methanol was used as blank, and ferrous sulfate was used as a standard reference. The results were expressed as μmol of Fe^2+^ equivalents (FE) per gram of DS (μmol FE/g DS).

#### 2.6.4. Hydrogen Peroxide Scavenging Assay (HPSA)

The HPSA was carried out following the method of Zhu [10] and Li [9]. In short, CFG extract (0.6 mL) was mixed with 0.9 mL H_2_O_2_ (400 mmol/L) and 1.5 mL of sodium phosphate buffer (45 mmol/L, pH 7.4). The reaction mixture was kept in the dark for 40 min, and then its absorbance was measured at 230 nm. The value of the HPSA was calculated from the standard curve for Trolox, and the percentage of H_2_O_2_ radical scavenging was evaluated using the following formula:

(3) $$\mathrm{Hydrogen}\;\mathrm{peroxide}\;\mathrm{scavenging}\;\left(\%\right)=\left[\left(A_{0}-A_{1}\right)/A_{0}\right]\times 100$$

where *A*_0_ is the absorbance of control and *A*_1_ is absorbance of the sample. The results were expressed as μmol of TE per g of DS (μmol TE/g DS).

### 2.7. Cell Assays for Antioxidative Activities

#### 2.7.1. Cell Culture

HepG2 cells were purchased from the cell bank of Sebachem (Shanghai, China). Cell culture was prepared following Bak [13] with certain modifications. First, HepG2 cells were cultured in DMEM medium supplemented with 1% penicillin-streptomycin (Fisher, Houston, TX, USA) and 10% fetal bovine serum. Then, all the cells were placed at 37 °C in a 5% CO_2_ incubator for 24~48 h. When the cell concentration reached 80%, the cells were digested with trypsin.

#### 2.7.2. Cell Viability Assay

Cell viability was performed on the previous method of Tan [14] with some modifications. Briefly, 2 × 10^4^ cells/well were seeded into a 96-well plate for 24 h. Then, the cells were treated with the CFG extract for 24 h. MTT solution (10 μL) was added to each well, and the cells were incubated at 37 °C in 5% CO_2_ for 24 h. Finally, the medium was removed, 150 μL DMSO was added, and the plate was gently shaken. The absorbance was measured at 517 nm.

Cell viability (%) was calculated with the equation:

(4) $$\mathrm{Cell}\;\mathrm{viability}\;\left(\%\right)=\left(A_{0}/A_{1}\right)\times 100\;$$

where *A*_0_ is the absorbance of the sample and *A*_1_ the absorbance of the blank.

### 2.8. Determination of Oxidative Stress Parameters

The cellular reactive oxygen species (ROS) levels were determined following a previous report [15] with some modifications. In brief, cells were seeded at a density of 2 × 10^4^ cells/well into 96-well plates, and cultured in a 37 °C, 5% CO_2_ incubator for 24 h. Then, 100 μL of no-toxic polyphenol extracts at the concentrations of 5, 10, 25 μg/mL was added into each well. Subsequently, 800 μmol/L H_2_O_2_ was added and incubated for 6 h. Finally, the cells were washed with PBS, and 10 μmol/L DCFH–DA (0.0125 mg/mL in medium without FBS) was added. The absorbance was determined at 488 (excitation wavelength) and 525 nm (emission wavelength) using a fluorescence enzyme labeler. (BioTek Synergy H1, Burlington, VT, USA).

The SOD, CAT, MDA activity and GSH levels were measured using the assay kits obtained from the Nanjing Jiancheng Institute of Biotechnology (Nanjing, China). All the procedures were carried out in accordance with the manufacturer’s instructions.

### 2.9. Data and Statistical Analysis

Data were presented as mean ± standard deviation (SD) of three replicates. To analyze the differences between the means of the treatment group and the control group, one-way ANOVA was applied to calculate the statistical significance. All graphs were generated using GraphPad Prism 8.0 (GraphPad, San Diego, CA, USA). The data were statistically analyzed using SPSS Version 18.0 software. (SPSS Inc., Chicago, IL, USA). *p* < 0.05 was regarded as the level of significance.

## 3. Results and Discussion

### 3.1. Total Phenolic Content (TPC) and Total Flavonoid Content (TFC)

The total phenolic content (TPC) and total flavonoid content (TFC) of the different extracts from CGF (FF and FP) are presented in Table 1.

Phenolic compounds are common secondary metabolites which are widely distributed in plants. Bamboo fruit, such as the fruit of *Melocanna baccifera*, is generally rich in nutrients and polyphenolic compounds [16]. As shown in Table 1, the TPC of SPs in FF and FP was 8.721 and 17.679 μmol FAE/g DS, respectively, and the TPC of IBPs in FF and FP was 7.544 and 12.903 μmol FAE/g DS, respectively. It can be observed that the TPC values of soluble fraction were higher than those of the insoluble fraction, whether in FF or FP of CFG, which is in agreement with previous studies of mistletoes [9] and red sorghums [17]. In addition, these results suggest that polyphenol molecules were more enriched in the FP than in the FF.

Flavonoids are the largest group of phenolic compounds. Increasing evidence indicates that flavonoids can possess antioxidant and anti-inflammatory effects [18,19]. From Table 1, it can be seen that the TFC of SP extracts was 1.237 and 1.052 CE/g DS in FF and FP, respectively, and the TFC of IBP extracts was 0.622 and 0.837 CE/g DS in FF and FP, respectively. In line with the TPC results, TFC of SPs was significantly higher than those of IBPs in two fractions of CFG fruit. The TFC of IBP extract in FP was higher than that of FF and similar to the TPC values in FP of FF and CFG. However, the TFC of SP extracts in FP was lower than that in FF, which may be due to the presence of different types of polyphenols in FF and FP or because SPs and IBPs in FF and FP can bind to polysaccharides on the cell wall with different affinities [20].

We can infer that the FP of CFG may contain more phenolic compounds due to the difference in polyphenol content between the FP and FF. Therefore, we decided to investigate the polyphenol composition of CFG fruit.

### 3.2. Identification of Phenolic Compositions

**Figure 1:**
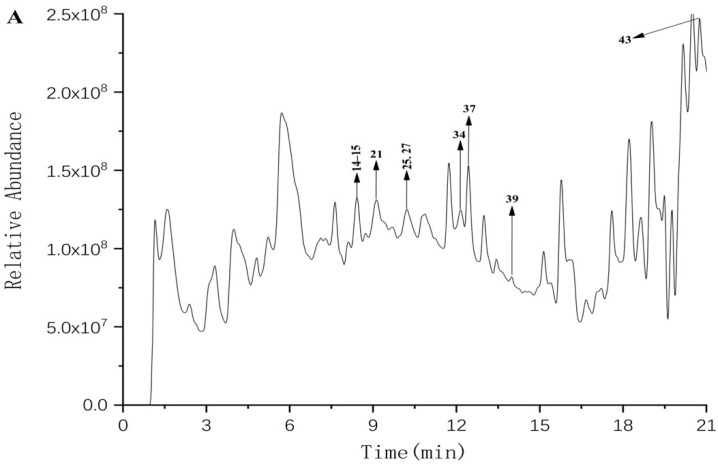

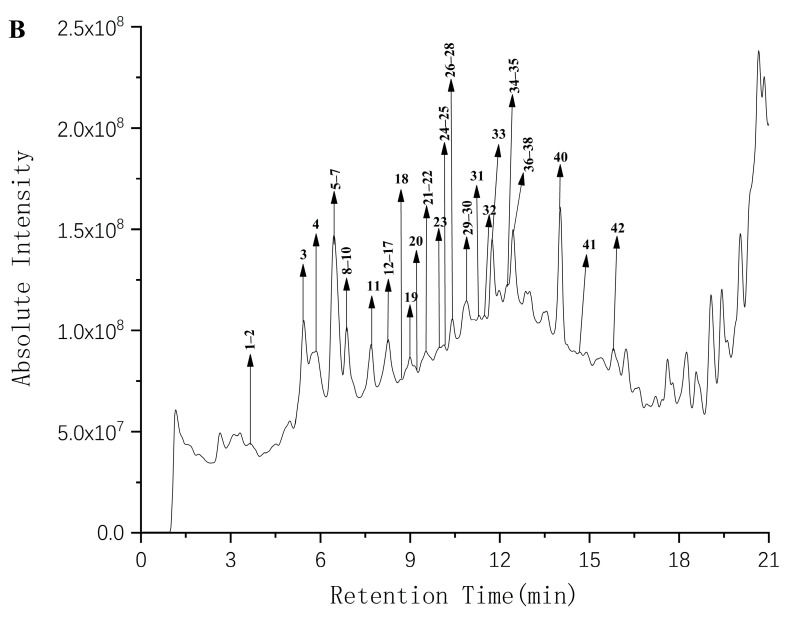
The total ion chromatogram The HPLC chromatograms of phenolic compounds in the FF (**A**) and FP (**B**) from CFG extracts are shown in Figure 1. The data were plotted using Origin software.

The polyphenols of FF and FP from CFG fruit were identified using UPLC–Q–TOF–MS/MS. Representative UPLC–QTOF–MS/MS total ion chromatograms (TIC) in the negative ion mode of polyphenols in CFG are shown in Figure 1. The phenolic compounds were tentatively identified by matching retention times (RT), *m*/*z* values, MS/MS fragments with compounds from the reported data in literature and database resources [21]. In addition, the relevant MS/MS spectra are provided in the Supplementary Materials.

A total of 43 compounds were initially identified in the negative mode. Table 2 lists the retention time (RT), molecular formulas, experimental molecular weights, and major fragment ions. Of these, 9 compounds were identified in the FF and 40 compounds in FP, with 6 of them present in both samples.

#### 3.2.1. Structural Characterization of Hydroxybenzoic Acid

Peak 1 had a molecular ion at *m*/*z* 181.0505, which fragmented into the fragment ion at *m*/*z* 151.0395 [M − CHO − H]^−^, and yielded another fragment ion at *m*/*z* 133.0292 [M − C_2_H_6_O_2_]^−^. It was tentatively identified as methyl vanillate based on the literature and database resources.

Peak 2 showed a [M − H]^−^ ion with an *m*/*z* of 151.0401 and yielded fragment ions at *m*/*z* 133.0302 [M − OH − H]^−^ and *m*/*z* 105.0344 [M − COOH − H]^−^, which were tentatively identified as vanillin.’

Peak 4 showed a [M − H]^−^ ion at *m*/*z* 211.0613, and the MS/MS spectrum showed fragment ions at *m*/*z* 193.0516 [M − OH − H]^−^ and 150.0313 [M − C_2_H_6_O_2_ − H]^−^, which were tentatively identified as methyl syringic acid.

Peak 7 showed a [M − H]^−^ ion at *m*/*z* 167.0348, which lost one molecule of CO_2_ to generate the fragment ion with *m*/*z* 123.0447, and lost one molecule of CH_3_ to generate another fragment ion at *m*/*z* 108.0214. Peak 7 was tentatively identified as vanillic acid.

Peak 9 showed a [M − H]^−^ peak with an *m*/*z* of 197.0455, and this ion lost one molecule of CO_2_ to generate the ion at *m*/*z* 153.0551, and lost one molecule of CH_3_ to generate a fragment ion *m*/*z* 182.0217. Peak 9 was tentatively identified as syringic acid.

Peak 10 showed a [M − H] ^−^ molecular ion at *m*/*z* 195.0661, which was generated through the loss of CH_2_O from the ion at *m*/*z* 165.0553, and then produced the ion at *m*/*z* 150.0319 by the further elimination of CH_3_. Peak 10 was tentatively identified as methyl veratrate.

Peak 17 showed a [M − H]^−^ molecular ion at *m*/*z* 165.0557, and the MS/MS spectrum showed fragment ions at *m*/*z* 150.0312 [M − CH_3_ − H]^−^ and 121.0284 [M − C_2_H_2_O − H]^−^. Peak 17 was tentatively identified as ethylvanillin.

#### 3.2.2. Structural Characterization of Flavonoids

Peak 3 presented the [M − H]^−^ ion at *m*/*z* 577.1357, and the MS/MS spectrum showed fragment ions at *m*/*z* 425.087 [M − C_8_H_8_O_3_ − H]^−^ and 287.056 [M − C_14_H_12_O_6_ − H]^−^. Peak 3 was tentatively identified as vitexin 2′′-O-p-coumarate.

Peak 6 showed a [M − H]^−^ molecular ion at *m*/*z* 289.0716, and the MS/MS spectrum showed fragment ions at *m*/*z* 137.0241 [M − C_8_H_8_O_3_ − H ]^−^ and 151.0396 [M − C_7_H_6_O_3_ − H]^−^. Peak 6 was tentatively identified as catechin.

Peak 11 showed a [M − H]^−^ ion at *m*/*z* 449.1456 with the MS/MS fragment ions at *m*/*z* 431.1346 [M − H_2_O − H]^−^ and 138.0319 [M − C_15_H_19_0_7_ − H]^−^. Compound 11 was tentatively identified as auriculoside.

As for peak 13, the [M − H]^−^ ion at *m*/*z* 257.0798 [M − CH_2_O − H]^−^ and 120.0217 [M − C_9_H_11_O_3_ − H]^−^ was obtained, and the MS/MS characteristic ion was presented at *m*/*z* 120.0217 [M − C_9_H_11_O_3_ − H]^−^. Peak 13 was tentatively identified as phloretin 4′-methyl ether.

Peak 23 showed the [M − H]^−^ ion with an *m*/*z* value of 313.1081, and the predominant fragment ions appeared at *m*/*z* 253.0868 [M − C_3_HO_2_ − H]^−^ and 266.0924 [M − CHO_2_ − H]^−^, which were tentatively identified as beta,2-Dihydroxy-4,6-dimethoxy-3-methylchalcone.

Peak 24 had a [M − H]^−^ ion with an *m*/*z* value of 593.1515, and its MS/MS spectrum showed fragment ions of *m*/*z* 181.051 [M − C_17_HO_11_ − H]^−^ and 315.0857 [M − C_9_HO_9_ − H]^−^. Peak 24 was tentatively identified as isoorientin 6′′-rhamnoside.

Peak 25 had a [M − H]^−^ ion at *m*/*z* 317.0664 and yielded an ion at *m*/*z* 125.0238 [M − C_10_H_8_O_4_ − H]^−^. Peak 25 was tentatively identified as dihydroisorhamnetin.

Peak 27 exhibited the [M − H]^−^ ion at *m*/*z* 609.1466 and fragmented at *m*/*z* 301.0346 [M − glc − rha − H]^−^ and 178.9986 [M − glc − rha − C_7_H_7_O_2_ − H]^−^. Peak 27 was tentatively identified as rutin.

Peak 33 had a [M − H]^−^ ion at *m*/*z* 337.1081 and yielded an ion at *m*/*z* 322.0826 [M − H − CH_3_ − H]^−^. Peak 33 was tentatively identified as psoralenol.

Peak 35 had a parent ion [M − H]^−^ at *m*/*z* 293.0819, and fragmented at *m*/*z* 189.0555 [M − CHO − H]^−^ and 119.0501 [M − CHO − H]^−^. Peak 35 was tentatively identified as 3-Methoxy-2-4H-1-benzopyran-4-one.

Peak 37 showed a parent ion [M − H]^−^ at *m*/*z* 271.0614, and fragmented at *m*/*z* 151.0039 [M − CHO − H]^−^ and 107.0142 [M − CHO − H]^−^. Peak 37 was tentatively identified as pinobanksin.

Peak 38 gave a [M − H]^−^ ion peak at *m*/*z* 417.1344 and presented the MS/MS fragment ion at *m*/*z* 387.1252 [M − CH_2_O − H]^−^, which was tentatively identified as cyclomulberrochromene.

#### 3.2.3. Structural Characterization of Coumarins

Peak 5 had a parent ion [M − H]^−^ at *m*/*z* 235.0609 and fragmented at *m*/*z* 205.0506 [M − CH_3_O − H]^−^. Peak 5 was tentatively identified as schinicoumarin.

Peak 14 had a [M − H]^−^ ion at *m*/*z* 163.0401 and formed an *m*/*z* of 119.0500 fragment ion when it lost CO_2_. Peak 14 was tentatively identified as p-coumaric acid.

Peak 16 had a [M − H]^−^ ion at *m*/*z* 161.0244, which shared the same fragment pattern with peak 14. Peak 16 was tentatively identified as 7-Hydroxycoumarin.

Peak 20 exhibited its [M − H]^−^ ion at *m*/*z* 327.1237 and produced two fragment ions at *m*/*z* 312.1015 [M − CH_3_O − H]^−^ and 281.0817 [M − C_2_H_6_O − H]^−^. Peak 20 was tentatively identified as decursin.

Peak 31 gave a [M − H]^−^ ion at *m*/*z* 267.066, and its MS/MS spectrum showed fragment ions at *m*/*z* 121.0292 [M − C_9_H_3_O_5_ − H]^−^ and 137.0245 [M − C_9_H_3_O_6_ − H]^−^. Peak 31 was tentatively identified as dalbergin.

Peak 40 showed a [M − H]^−^ ion with an *m*/*z* value of 219.0661, and the MS/MS spectrum showed ions at *m*/*z* 203.0355 [M − CH_3_ − H]^−^ and 204.0423 [M − CH_4_ − H]^−^. Peak 40 was tentatively identified as polygonolide.

Peak 42 gave the [M − H] ^−^ ion with an *m*/*z* of 243.1026 and fragmented into MS/MS fragment ion at *m*/*z* 199.1134 [M − CO_2_ − H]^−^ and 227.1081 [M − O − H]^−^. Peak 42 was tentatively identified as osthole.

#### 3.2.4. Structural Characterization of Cinnamic Acids

Peak 8 showed a [M − H] ^−^ ion at *m*/*z* 271.0614 and fragmented at *m*/*z* 135.0446 [M − CH_2_O_2_ − H]^−^ and 133.0296 [M − CH_4_O_2_ − H]^−^. Peak 8 was tentatively identified as caffeic acid.

Peak 12 had a [M − H]^−^ ion at *m*/*z* 193.0504 and presented the typical fragment at *m*/*z m*/*z* 178.0271 [M − CH_3_ − H]^−^ and 108.0219 [M − CH_3_ − C_3_H_2_O_2_ − H]^−^. Peak 12 was tentatively identified as ferulic acid.

Peak 15 had a [M − H]^−^ ion at *m*/*z* 367.1036 and fragmented at *m*/*z* 134.0372 [M − C_9_H_13_O_7_ − H]^−^ and 193.0504 [M − C_7_H_10_O_5_ − H]^−^. Based on the literature, it was tentatively identified as 5-O-feruloylquinic acid.

Peak 19 had a [M − H] ^−^ ion at *m*/*z* 563.1409, and presented the MS/MS characteristic ions at *m*/*z* 353.0668 [M − C_7_H_14_O_7_ − H]^−^ and 443.099 [M − C_4_H_8_O_4_ − H]^−^. Peak 19 was tentatively identified as yopaaoside B.

Peak 21 had a [M − H]^−^ ion at *m*/*z* 223.0611 and presented the MS/MS characteristic ions at *m*/*z* 208.038 [M − CH_3_ − H]^−^ and 164.0475 [M − CH_3_ − CO_2_ − H]^−^. Peak 21 was tentatively identified as sinapic acid.

Peak 22 gave a molecular ion [M − H] ^−^ at *m*/*z* 177.0555 and showed a fragment ion at *m*/*z* 162.0314, corresponding to the loss of a CH_3_ residue. Peak 22 was tentatively identified as methyl 4-hydroxycinnamate.

Peak 32 had a molecular ion at *m*/*z* 371.1129, corresponding to the molecular formula C_20_H_20_O_7_. Two major fragment ions were observed at *m*/*z* 283.0976 [M − C_3_H_4_O_3_ − H]^−^ and 162.0319 [M − C_11_H_13_O_4_ − H]^−^. Peak 32 was tentatively identified as cimicifugic acid.

Peak 34 showed a molecular ion at *m*/*z* 147.045, and the MS/MS fragmentation with an ion at *m*/*z* 103.055 corresponded to the loss of CO_2_. Peak 34 was tentatively identified as cinnamic acid.

Peak 39 had a molecular ion at *m*/*z* 637.2141, which yielded an ion at *m*/*z* 387.1252 [M − OCH_2_ − H]^−^. Peak 39 was tentatively identified as leucosceptoside A.

Peak 43 had a [M − H]^−^ ion at *m*/*z* 585.4876 and yielded an ion at *m*/*z* 281.2483 [M − C_20_H_32_O_2_ − H]^−^. Peak 43 was tentatively identified as erythrinasinate A.

#### 3.2.5. Structural Characterization of Terpenoid

Peak 18 showed a [M − H]^−^ ion with an *m*/*z* of 409.1294 and presented the MS/MS spectrum showing fragment ions at *m*/*z* 361.1084 [M − CH_4_O_2_ − H]^−^ and 121.0295 [M − C_16_H_16_O_5_ − H]^−^. Peak 18 was tentatively identified as lactucopicrin.

#### 3.2.6. Structural Characterization of Lignin

Peak 26 had a [M − H]^−^ ion at *m*/*z* 373.1291, and fragmented at *m*/*z* 179.0709 [M − C_10_H_10_O_4_ − H]^−^ and 194.0566 [M − C_10_H_11_O_3_ − H]^−^. Peak 26 was tentatively identified as nortrachelogenin.

Peak 28 had a molecular ion at *m*/*z* 433.1501, corresponding to the molecular formula of C_22_H_26_O_9_. Two major fragment ions were observed at *m*/*z* 403.1401 [M − CHO_2_ − H]^−^ and 373.1297 [M − C_2_H_4_O_2_ − H]^−^. Peak 28 was tentatively identified as ciwujiatone.

Peak 29 had a deprotonated molecular ion peak at *m*/*z* 339.1239, which was generated by continuously losing two molecules of CH_3_ to produce ions at *m*/*z* 324.1004 [M − CH_3_ − H]^−^ and 309.0763 [M − 2CH_3_ − H]^−^, respectively. Peak 29 was tentatively identified as futoenone.

Peak 30 had a molecular ion at *m*/*z* 195.0661, fragmented into the fragment ion at *m*/*z* 150.0319 [M − C_22_H_27_O_7_ − H]^−^, and yielded another fragment ion at *m*/*z* 165.0552 [M − C_21_H_24_O_7_ − H]^−^. Peak 30 was tentatively identified as lappaol C.

Peak 36 had a molecular ion at *m*/*z* 297.1133, corresponding to the molecular formula of C_18_H_18_O_4_. Two major fragment ions were observed at *m*/*z* 107.05 [M − C_11_H_10_O_3_ − H]^−^ and 253.1234 [M − CO_2_ − H]^−^. Peak 36 was tentatively identified as enterolactone.

Peak 41 showed a molecular ion at *m*/*z* 325.1082 with a chemical composition of C_19_H_18_O_5_. The predominant fragment ions appeared at *m*/*z* 310.0841 [M − CH_3_ − H]^−^ and 281.0808 [M − CH_3_ − CHO − H]^−^. Peak 41 was tentatively identified as ailanthoidol.

#### 3.2.7. Analysis of UPLC–QTOF–MS/MS

Generally, we found that the phenolic compounds detected in the present study were also found in bamboo fruits of *Melocanna baccifera* [61], including cinnamic acid and syringic acid. Cinnamic acid is a phenolic compound naturally occurring in various vegetables, seeds, and also enriched in daily diets [62]. In addition, syringic acid is a phenolic compound that acts as a free radical scavenging antioxidant in pharmacology [63] and is rich in many edible mushrooms and vegetables and food and beverage plants [64]. The presence of polyphenols may be responsible for their antioxidant activities. Hence, the phenolic compounds in CFG fruits have potential for further research.

### 3.3. In Vitro Antioxidant Activities

The results of four in vitro antioxidant capacity evaluation tests are shown in Table 3.

DPPH radical scavenging capacity assay was used for assessing the hydrogen atom or electron donor capacity of phenolic compounds. As shown in Table 3, the DPPH radical scavenging of SPs in FF and FP was 1.355 and 4.686 μmol TE/g DS, and that of IBPs was 1.124 and 1.292 μmol TE/g DS. Thus, DPPH scavenging activity of SPs was higher than that of IBPs. Aside from DRSA, the FRAP of the FF and FP extracts of CFG was also determined in this study. The FRAP of SPs was 11.098 μmol FE/g DW in FF and 17.424 μmol FE/g DW in FP, while that for IBPs was 6.433 μmol FE/g DW in FF and 10.597 μ mol FE/g DW in FP. In addition, the TEAC of SPs was 35.328 μmol TE/g DS in FF and 59.847 μmol TE/g DS in FP, while that for IBPs was 17.758 μmol FE/g DW in FF and 56.299 μmol TE/g DS in FP. The HPSA results of SPs in FF and FP were 47.547 and 72.884 μmol TE/g DS, and those of IBPs were 39.281 and 64.843 μmol TE/g DS. In short, like DPPH radical scavenging ability, the polyphenols in FP showed a more robust antioxidant capacity in the TEAC, HPSA and FRAP assays than in FF of CFG. The SP extract in CFG also had significantly higher antioxidant capacities in terms of FRAP, HPSA, DRSA and TEAC than the IBP extracts (*p* < 0.05). These findings are similar to those reported in previous studies on other plants, such as mistletoe [9] and L. macranthoides [65].

Thus, the in vitro antioxidant test results suggested that CFG fruit had antioxidant properties, and the FP with higher polyphenol content had more vigorous antioxidant activities than the FF.

### 3.4. Cell Viability

Since the fruit of CFG exhibited notable antioxidant activity in vitro, the inner effect on cell levels required further study. Cell viability is often employed as an indicator of cytotoxicity [66], and the cytotoxic effects in FF and FP of CFG fruit on the HepG2 cells were evaluated with the MTT assay. Cytotoxicity was considered when the cell viability was less than 90%.

In this study, H_2_O_2_ was used to induce oxidative stress injury in HepG2 cells and to assess the protective effect of polyphenols in CFG.

**Figure 2:**
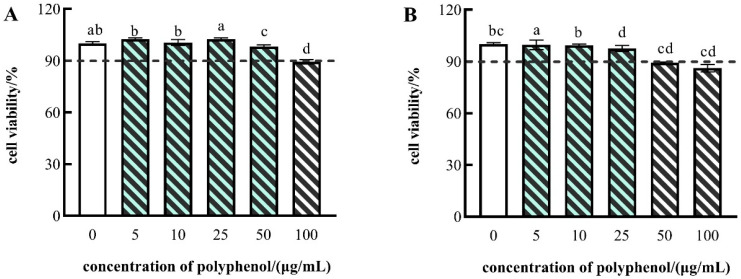
Cell viability of HepG2 cells treated with different concentrations of FF polyphenol (**A**) and FP polyphenol (**B**). The data are presented as the mean ± SD (*n* = 3). Different lowercase letters indicate significance at *p* < 0.05.

As shown in Figure 2, the polyphenols in FP showed no cytotoxicity at polyphenol concentrations of 5, 10, or 25 μg/mL, respectively, and the FF showed no cytotoxicity at polyphenol concentrations of 5, 10, 25, or 50 μg/mL. Consequently, to ensure that the polyphenol concentrations remained consistent, the polyphenol concentrations of 5, 10, and 25 μg/mL were employed for subsequent experiments.

### 3.5. Protective Effects of Polyphenols from CFG on H^2^O^2^-Induced Intracellular ROS Production in HepG2 Cells

**Figure 3:**
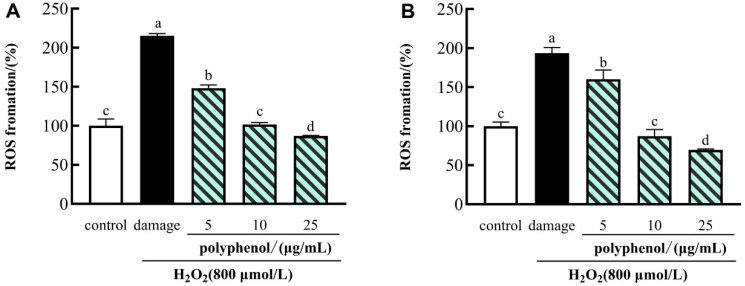
Effects of polyphenol (FF (**A**) and FP (**B**)) from CFG on the levels of ROS in oxidative damage HepG2 cells. The data were presented as the mean ± SD (*n* = 3). Values with different lowercase letters were significantly different at *p*  <  0.05.

The effects of the polyphenol on H_2_O_2_-induced ROS generation in HepG2 cells are shown in Figure 3. There were 40–60% viable cells in the presence of 800 μmol/L H_2_O_2_ compared to control cells. Therefore, in the following experiments, 800 μmol/L of H_2_O_2_ treatment for 24 h was used to induce HepG2 cell injury. Compared with the control group, the levels of intracellular ROS in HepG2 cells were prominently increased after H_2_O_2_ induction (Figure 3). Those results showed that the increased intracellular ROS levels caused by H_2_O_2_-induction were attenuated in the HepG2 cells pretreated with polyphenols. Among them, compared with those in control cells, the intracellular ROS levels were decreased from 215.152% to 87.147% with increasing polyphenol concentrations of FF. Similarly, we observed that the ROS levels were reduced by the treatment of the polyphenol in FP from 193.575% to 69.575%. The same trends of ROS production were observed in *Tamarindus indica* leaf extract [67] and resveratrol [68]. Our results indicated that the effects of polyphenols were more prominent at the highest concentration (25 μg/mL) than at the lowest concentration (5 μg/mL), both in the FF and FP. Hence, we can conclude that the polyphenols of FF and FP in CFG could protect cells from damage imposed by ROS.

### 3.6. The Effects of CFG on the Activities of SOD, CAT, GSH and MDA in H_2_O_2_-Induced HepG2 Cells

SOD and CAT are critical antioxidant enzymes that can play an essential role in oxygen metabolizing cells. SOD can convert superoxide to H_2_O_2_, which is further converted via CAT into H_2_O and O_2_ [69], and the SOD activity levels indirectly reflect the body’s ability to scavenge oxygen free radicals [70]. In addition, reduced glutathione (GSH) is an important intracellular antioxidant that can scavenge H_2_O_2_ in favor of scavenging oxidants [71]. Malondialdehyde (MDA), a byproduct of lipid peroxidation, is widely used as a crucial indicator for oxidative stress [15]. To investigate the protective effects of polyphenols from CFG on H_2_O_2_-induced cell injury in HepG2 cells, the SOD and CAT activities, GSH and MDA levels were measured using commercial kits.

**Figure 4:**
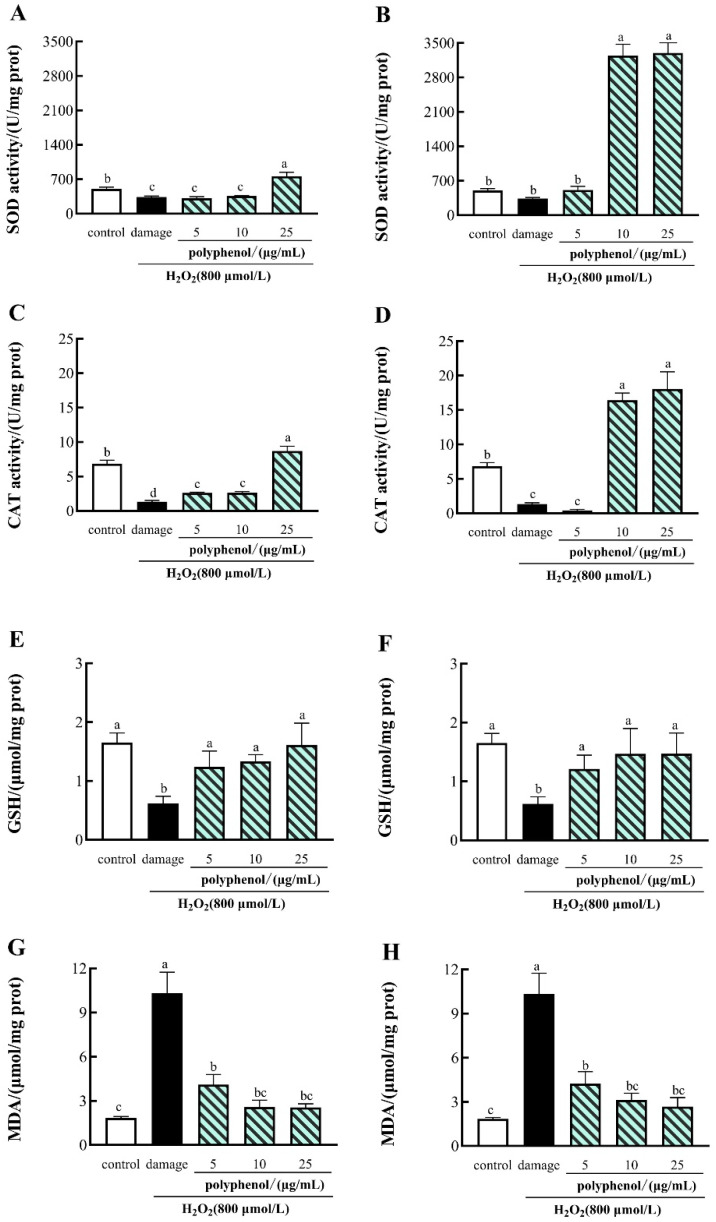
(**A**) Effect of FF polyphenol on intracellular SOD. (**B**) Effect of FP polyphenol on intracellular SOD. (**C**) Effect of FF polyphenol on CAT activity. (**D**) Effect of FP polyphenol on CAT activity. (**E**) Effect of FF polyphenol on GSH. (**F**) Effect of FP polyphenol on GSH. (**G**) Effect of FF polyphenol on MDA activity. (**H**) Effect of FP polyphenol on MDA activity. All data are presented as the mean ± SD (*n* =3). Values with different lowercase letters are significantly different at *p*  <  0.05.

As shown in Figure 4, compared with the control group, the activities of SOD, CAT and GSH were dramatically decreased by H_2_O_2_ in the HepG2 cells and substantially increased the levels of MDA in H_2_O_2_-treated HepG2 cells. Treatment of 25 μg/mL of FF polyphenols and 10, 25 μg/mL of FP polyphenols significantly increased the activities of SOD and CAT, and the treatment of 5,10 and 25 μg/mL of FF and FP polyphenols significantly increased the activities of GSH, compared with the H_2_O_2_-treated damage group. In contrast, the treatment of 5, 10 and 25 μg/mL FF and FP polyphenols significantly reduced the MDA levels in H_2_O_2_-induced HepG2 cells (*p*  <  0.05). Similar results have been reported in *Myrica rubra* Bark [72].

The results indicated that the polyphenols of the FF and FP from CGF could exert protective action against H_2_O_2_-induced oxidative damage to HepG2 cells, especially at a high concentration.

## 4. Conclusions

The phenolic compounds in CFG fruits were identified for the first time using UPLC/Q–TOF–MS/MS. A total of 43 phenolic compounds were identified, including 7 hydroxybenzoic acids, 12 flavonoids, 7 coumarins, 10 hydroxycinnamic acids, 1 terpenoid, and 6 lignans. The antioxidant activities of phenolic compounds in CFG were reported for the first time. Moreover, the SP and IBP extract contents and the in vivo and in vitro antioxidant activity in FF and FP were compared. The results showed that the TPC of SPs and IBPs in FP was significantly higher than that in FF. The SP extracts in FP showed higher antioxidant activity compared to those in FF. In addition, the polyphenol extracts of FF and FP from CFG protected against H_2_O_2_-induced oxidative stress in HepG2 cells. Therefore, this study provides a basis for further research on CFG fruits and a scientific basis for the exploitation of CFG.

## Figures and Tables

**Table 1:** Results of total polyphenol and flavonoid content in soluble conjugated and insoluble bound phenolic of CGF.

Phenol Analyses	Phenolics	FF	FP
TPC (μmol FAE/g DS)	SPs	8.721 ± 0.499 ^b^	17.679 ± 0.550 ^a^
	IBPs	7.544 ± 0.592 ^b^	12.903 ± 0.480 ^a^
TFC (μmol CE/g DS)	SPs	1.237 ± 0.027 ^a^	1.052 ± 0.048 ^b^
	IBPs	0.622 ± 0.022 ^b^	0.837 ± 0.047 ^a^

SPs, soluble polyphenols; IBPs, insoluble-bound polyphenols; TPC total polyphenol content; TFC, total flavonoid content; FF, fruit flesh; FP, fruit pulp; Data are expressed as mean ± SD. The different superscripted small letters in each row indicate significant differences (*p* < 0.05, *n* = 3). FAE, ferulic acid equivalents; CE, catechin equivalents; DS, dry sample.

**Table 2:** Phenolic compounds identified in FP and FF from CFG by UPLC–Q–TOF–MS/MS in negative mode.

Peak No.	RT (min)	Formula	Exact Mass, [M − H]^−^, *m*/*z*	Theoretical Mass, [M − H]^−^, *m*/*z*	Error (ppm)	Characteristic MS/MS Ions (*m*/*z*)	Tentative Identification	Plant Part	Reference	Database		
										TCM	Metlin	PubChem
1	4.288	C_9_H_10_O_4_	181.0505	181.0506	0.7	151.0396	Methyl vanillate	FP	[22]		√	√
						133.0292						
2	4.295	C_8_H_8_O_3_	151.0401	151.0401	−0.2	105.0344	Vanillin	FP	[23]	√	√	√
						133.0302						
3	5.536	C_30_H_26_O_12_	577.1357	577.1351	−1	425.087	Vitexin 2′′-O-p-coumarate	FP	[24]	√	√	√
						287.056						
4	5.961	C_10_H_12_O_5_	211.0613	211.0612	−0.5	150.0313	Methyl Syringic acid	FP	[25]			√
						193.0516						
5	6.083	C_12_H_12_O_5_	235.0609	235.0612	0.1	205.0506	Schinicoumarin	FP	[26]	√		√
6	6.127	C_15_H_14_O_6_	289.0716	289.0718	0.6	137.0241	Catechin	FP	[27]		√	√
						151.0396						
7	6.361	C_8_H_8_O_4_	167.0348	167.035	1.1	108.0215	Vanillic acid	FP	[28]	√	√	√
						123.0448						
8	6.745	C_9_H_8_O_4_	179.0348	179.035	1	135.0446	Caffeic acid	FP	[29]	√	√	√
						133.0296						
9	6.985	C_9_H_10_O_5_	197.0455	197.0455	0.2	182.0217	Syringic acid	FP	[30]	√	√	√
						153.0551						
10	7.156	C_10_H_12_O_4_	195.0661	195.0663	0.1	150.0319	Methyl veratrate	FP	[31]	√		√
						165.0554						
11	7.556	C_22_H_26_O_10_	449.1456	449.1453	−0.6	431.1346	Auriculoside	FP	[32]		√	√
						138.0319						
12	8.052	C_10_H_10_O_4_	193.0504	193.0506	1.2	178.0271	Ferulic acid	FP	[33]	√	√	√
						108.0219						
13	8.074	C_16_H_16_O_5_	287.0924	287.0925	0.3	120.0217	Phloretin 4′-methyl ether	FP	[34]			√
14	8.23	C_9_H_8_O_3_	163.0401	163.0401	−0.2	119.05	p-coumaric acid	FP, FF	[35]	√	√	√
15	8.34	C_17_H_20_O_9_	367.1036	367.1035	−0.4	193.0504	5-O-Feruloylquinic acid	FF	[36]	√	√	√
						134.0372						
16	8.489	C_9_H_6_O_3_	161.0244	161.0244	0.1	134.0366	7-Hydroxycoumarin	FP	[37]	√		√
						119.05						
17	8.63	C_9_H_10_O_3_	165.0557	165.0557	0.1	150.0312	Ethylvanillin	FP	[38]	√	√	√
						121.0284						
18	8.647	C_23_H_22_O_7_	409.1294	409.1293	0.3	121.0295	Lactucopicrin	FP	[39]	√	√	√
						361.1084						
19	8.865	C_26_H_28_O_14_	563.1409	563.1406	1.3	353.0668	Yopaaoside B	FP	[40]	√		√
						443.099						
20	8.931	C_19_H_20_O_5_	327.1237	327.1238	0.3	281.0817	Decursin	FP	[41]	√	√	√
						312.1015						
21	9.007	C_11_H_12_O_5_	223.0611	223.0612	0.4	208.038	Sinapic acid	FP, FF	[42]	√	√	√
						164.0475						
22	9.324	C_10_H_10_O_3_	177.0555	177.0557	1.2	162.0314	Methyl 4-hydroxycinnamate	FP	[43]	√		√
23	9.679	C_18_H_18_O_5_	313.1081	313.1081	0.2	253.0868	beta,2-Dihydroxy-4,6-dimethoxy-3-methylchalcone	FP	[44]		√	√
						266.0924						
24	9.997	C_27_H_30_O_15_	593.1515	593.1512	−0.5	181.051	Isoorientin 6′′-rhamnoside	FP	[45]		√	√
						315.0857						
25	10.226	C_16_H_14_O_7_	317.0664	317.0667	0.9	125.0238	Dihydroisorhamnetin	FP, FF	[46]		√	√
26	10.36	C_20_H_22_O_7_	373.1291	373.1293	−0.8	179.0709	Nortrachelogenin	FP	[47]	√		√
						194.0566						
27	10.372	C_27_H_30_O_16_	609.1466	609.1461	−0.8	301.0346	Rutin	FP, FF	[48]	√	√	√
						178.9986						
28	10.498	C_22_H_26_O_9_	433.1501	433.1504	1.7	403.1401	Ciwujiatone	FP	[49]	√		√
						373.1297						
29	10.776	C_20_H_20_O_5_	339.1239	339.1238	0.5	324.1004	Futoenone	FP	[50]	√		√
						309.0763						
30	10.785	C_30_H_34_O_10_	553.2079	553.2079	0	165.0552	Lappaol C	FP	[51]		√	√
						150.0319						
31	10.895	C_16_H_12_O_4_	267.066	267.0663	−1	137.0245	Dalbergin	FP	[52]	√	√	√
						121.0292						
32	10.985	C_20_H_20_O_7_	371.1129	371.1136	−2.6	283.0976	Cimicifugc acid	FP		√		√
						162.0319						
33	11.495	C_20_H_18_O_5_	337.1081	337.1081	0.1	322.0826	Psoralenol	FP	[53]	√	√	√
34	12.069	C_9_H_8_O_2_	147.045	146.0373	1	103.055	Cinnamic acid	FP, FF	[54]	√	√	√
35	12.188	C_18_H_14_O_4_	293.0819	293.0819	0.1	189.0555	3-Methoxy-2-4H-1-benzopyran-4-one	FP			√	√
						119.0501						
36	12.484	C_18_H_18_O_4_	297.1133	297.1132	0.4	107.05	Enterolactone	FP	[55]	√	√	√
						253.1234						
37	12.625	C_15_H_12_O_5_	271.0614	271.0612	−0.7	151.0039	Pinobanksin	FP, FF	[56]	√	√	√
						107.0142						
38	12.688	C_25_H_22_O_6_	417.1344	417.1344	−4.8	387.1252	Cyclomulberrochromene	FP	[57]	√		√
						399.1254						
39	13.896	C_30_H_38_O_15_	637.2141	637.2138	−0.5	515.1785	Leucosceptoside A	FF	[58]	√		√
40	14.097	C_12_H_12_O_4_	219.0661	219.0663	0.9	204.0423	Polygonolide	FP		√	√	√
						203.0355						
41	14.41	C_19_H_18_O_5_	325.1082	325.1081	6.3	310.0841	Ailanthoidol	FP	[59]	√		√
						281.0808						
42	15.821	C_15_H_16_O_3_	243.1026	243.1027	0.3	227.1081	Osthole	FP	[60]	√	√	√
						199.1134						
43	20.942	C_38_H_66_O_4_	585.4876	585.4888	2.1	281.2483	Erythrinasinate A	FF			√	√

Based on the structural characteristics, the identified compounds included 7 hydroxybenzoic acids (Peaks 1, 2, 4, 7, 9, 10, 17), 12 flavonoids (Peaks 3, 6, 11, 13, 23, 24, 25, 27, 33, 35, 37, 38), 7 coumarins (Peaks 5, 14, 16, 20, 31, 40, 42), 10 Hydroxycinnamic acids (Peaks 8, 12, 15, 19, 21, 22, 32, 34, 39, 43), 1 terpenoid (Peak 18), and 6 lignins (Peaks 26, 28, 29, 30, 36, 41).

**Table 3:** Total polyphenols content and the in vitro antioxidant activity of CFG polyphenol extract (*n* = 3).

DPPH Radical Scavenging Activity (μmol TE/g DS)		
Plant Material	SPs	IBPs
FF	1.355 ± 0.018 ^Ay^	1.124 ± 0.080 ^By^
FP	4.686 ± 0.126 ^Ax^	1.292 ± 0.137 ^Bx^
**Ferric Reducing Antioxidant Power (μmol FE/g DS)**		
Plant Material	SPs	IBPs
FF	11.098 ± 0.708 ^By^	6.433 ± 0.324 ^Ax^
FP	17.424 ± 0.353 ^Ax^	10.597 ± 0.369 ^Ay^
**Trolox Equivalent Antioxidant Capacity (μmol TE/g DS)**		
Plant Material	SPs	IBPs
FF	35.328 ± 2.819 ^Ay^	17.758 ± 1.234 ^By^
FP	59.847 ± 0.371 ^Ax^	56.299 ± 0.241 ^Bx^
**Hydrogen Peroxide Scavenging Activity (μmol TE/g DS)**		
Plant Material	SPs	IBPs
FF	47.547 ± 0.967 ^Ay^	39.281 ± 0.796 ^By^
FP	72.884 ± 1.924 ^Ax^	64.843 ± 1.138 ^Bx^

FF, fruit flesh; FP, fruit pulp; SPs, soluble polyphenol; IBPs, insoluble-bound polyphenol; FE, Fe^2+^ equivalents; TE, Trolox equivalents; DS, Dry sample; Values are mean ± standard deviation (*n* = 9); Values in each row having the different big letter superscripts are significantly different (*p* < 0.05); Values in the same column with different small letter superscripts mean significant difference (*p* < 0.05).

## Data Availability

Not applicable.

## Supplementary Materials

The following supporting information can be downloaded at: https://www.mdpi.com/article/10.3390/molecules27123767/s1. Figure S1–S43: relevant MS/MS spectra.
