# Supplementary figures and images for: UPLC–Q–TOF–MS/MS Analysis of Phenolic Compounds from the Fruit of Cephalostachyum fuchsianum Gamble and Their Antioxidant and Cytoprotective Activities
Source: Molecules. 2022 Jun 11;27(12):3767. doi: 10.3390/molecules27123767 (PMC9227481; doi:10.3390/molecules27123767)

1

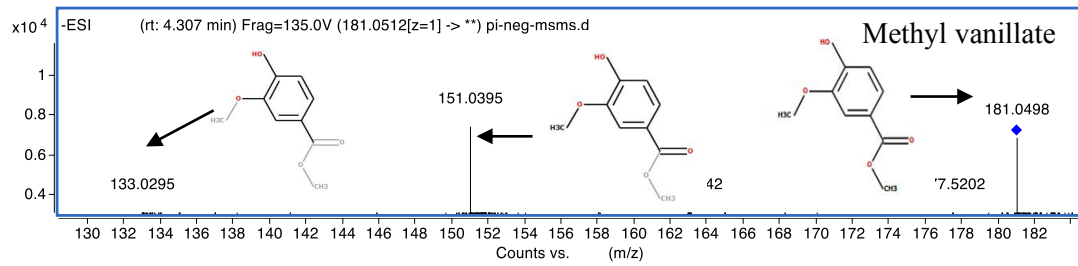

2

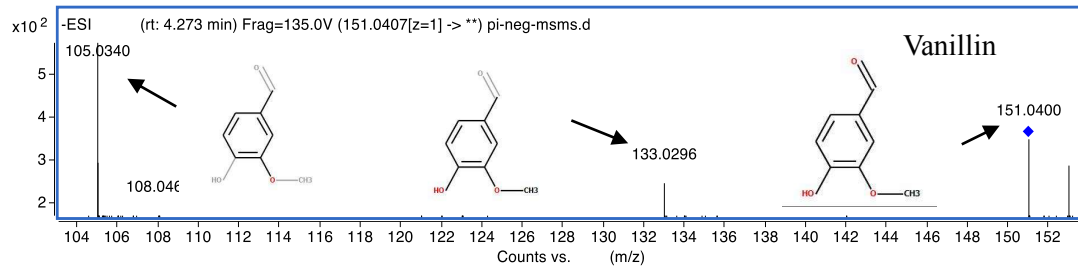

3

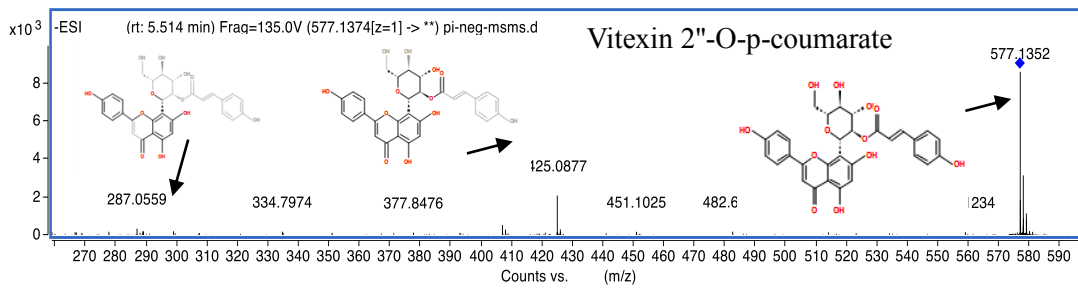

4

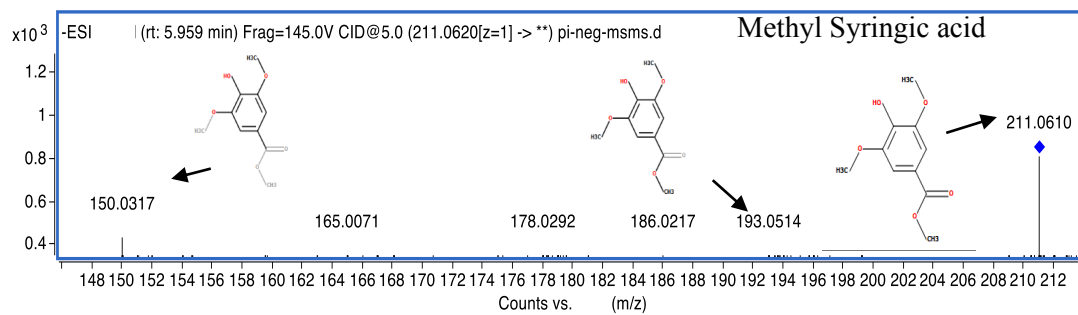

5

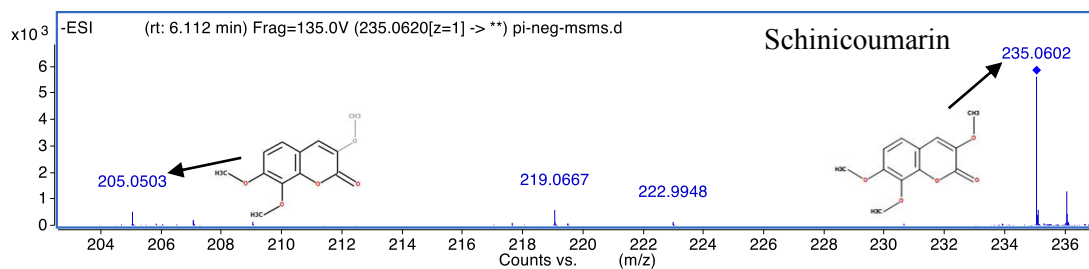

6

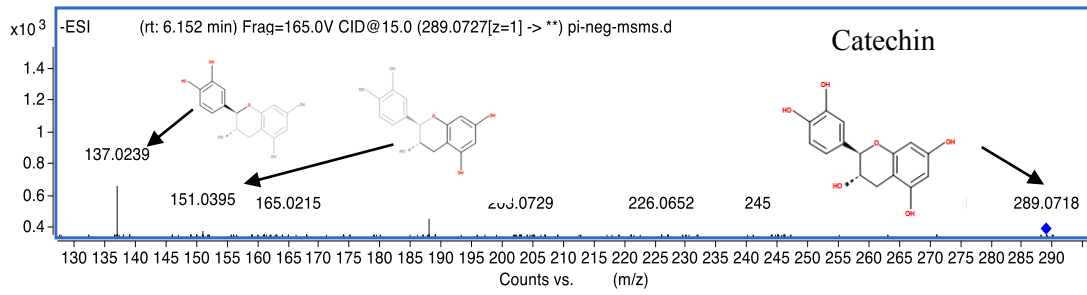

7

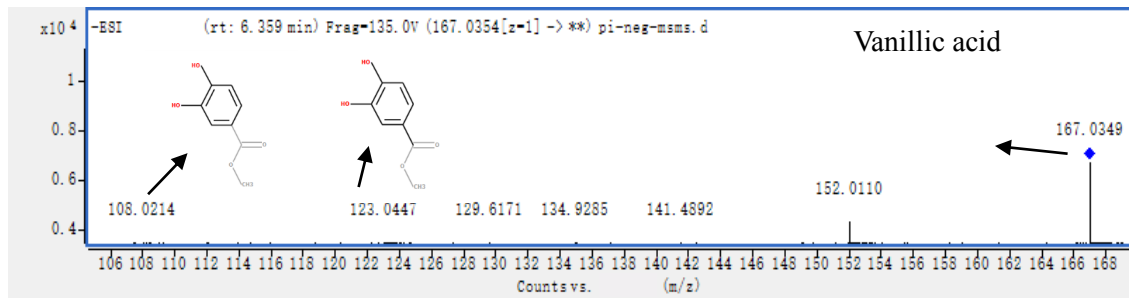

8

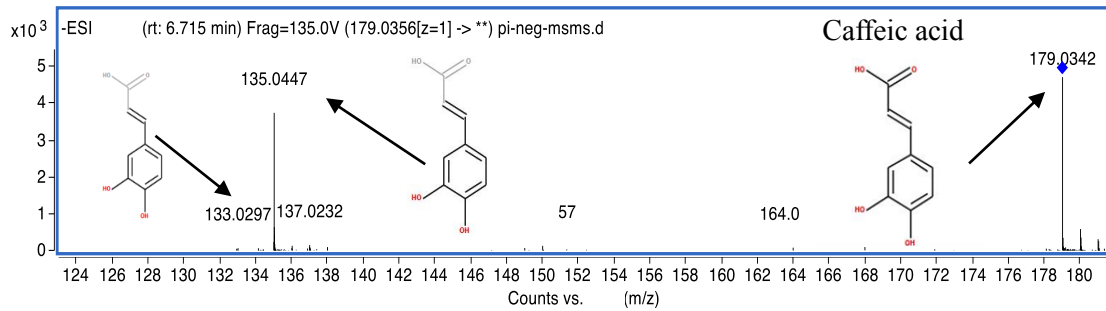

9

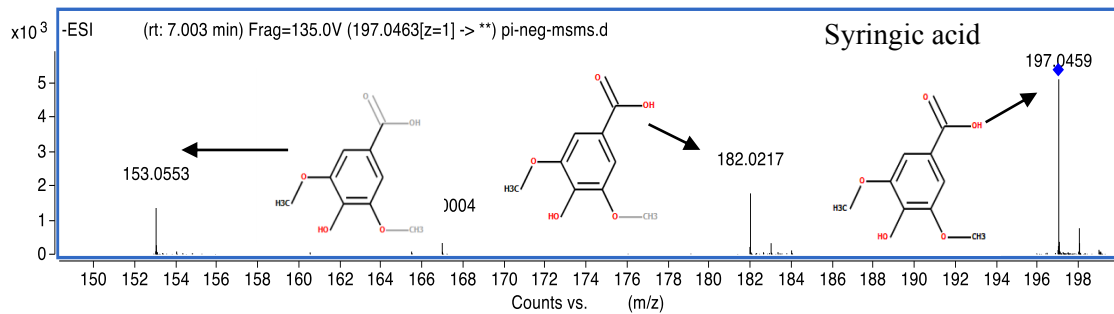

10

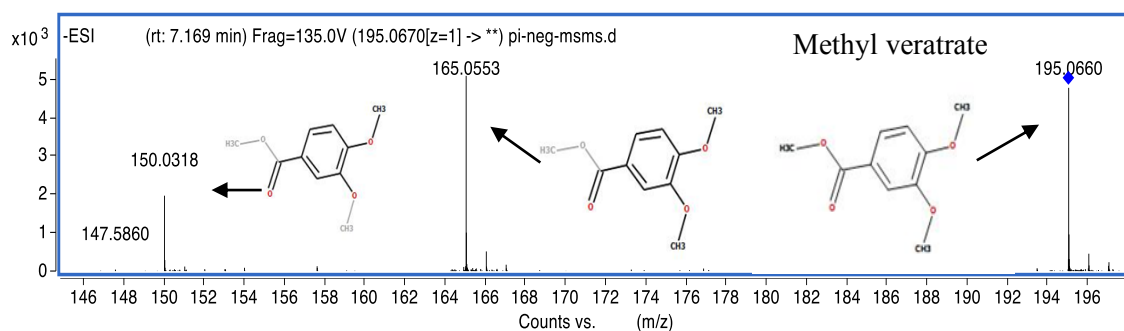

11

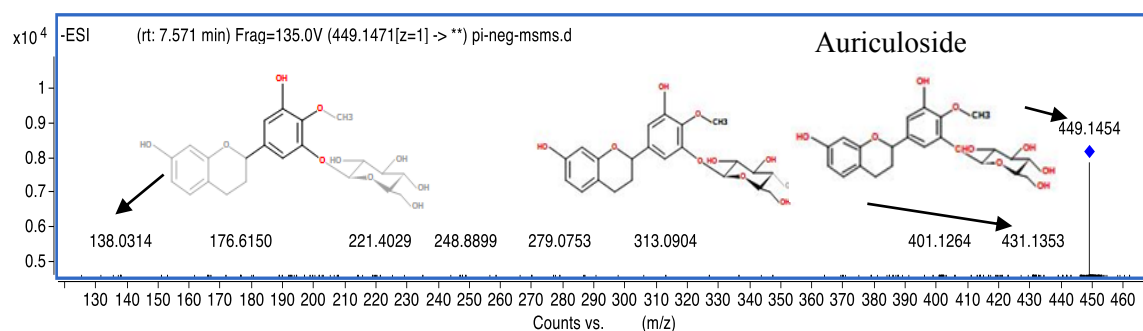

12

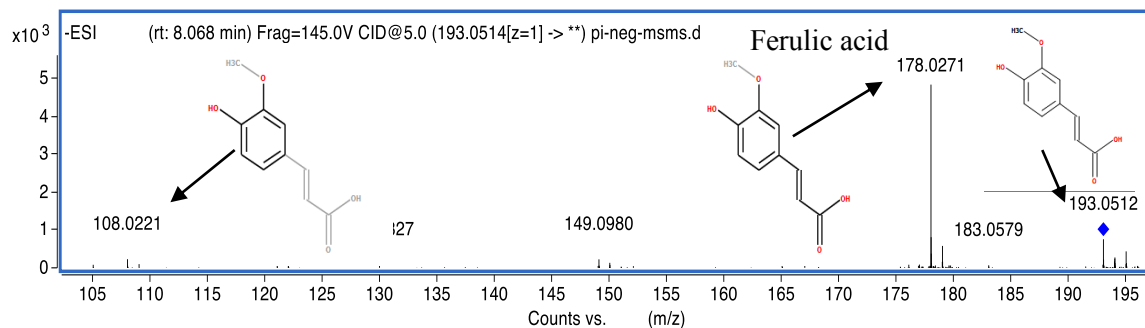

13

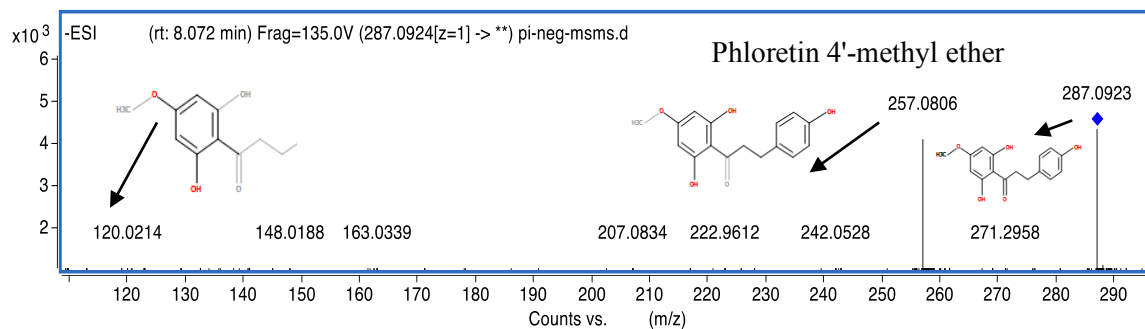

14

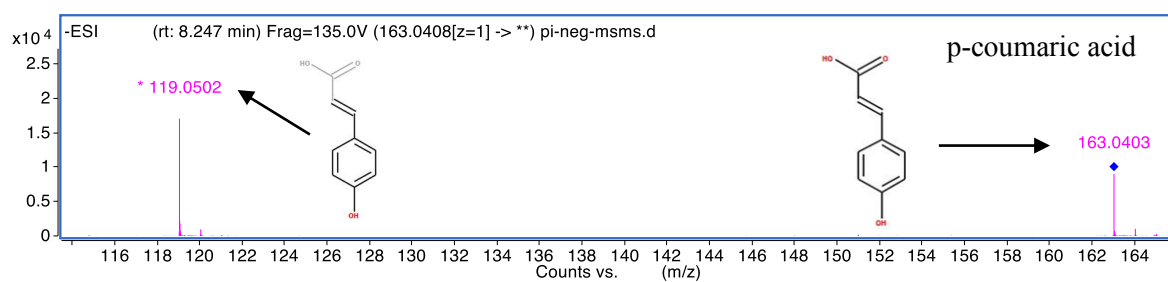

15

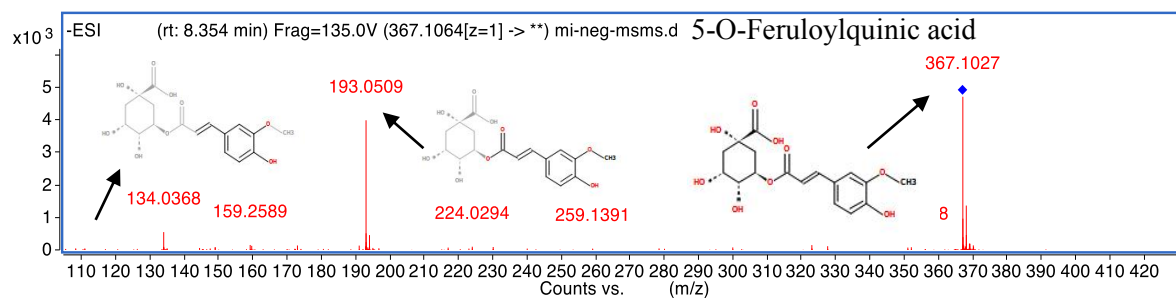

16

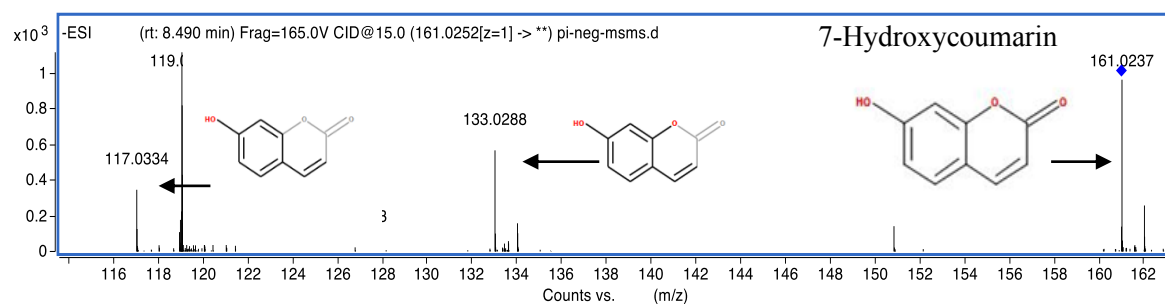

17

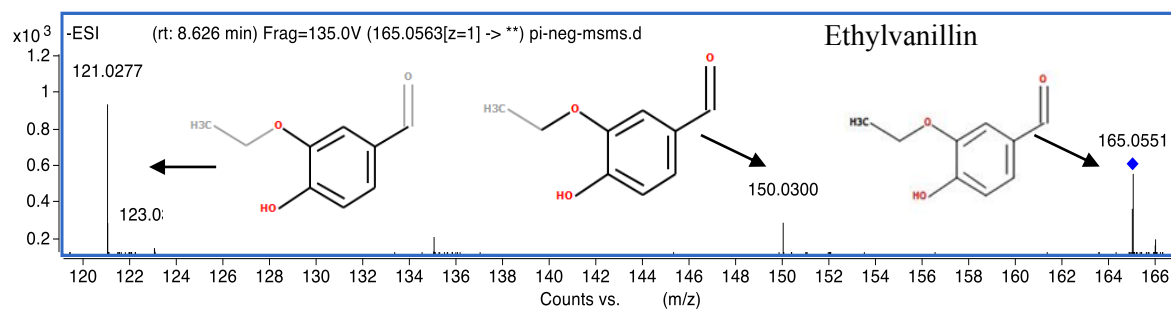

18

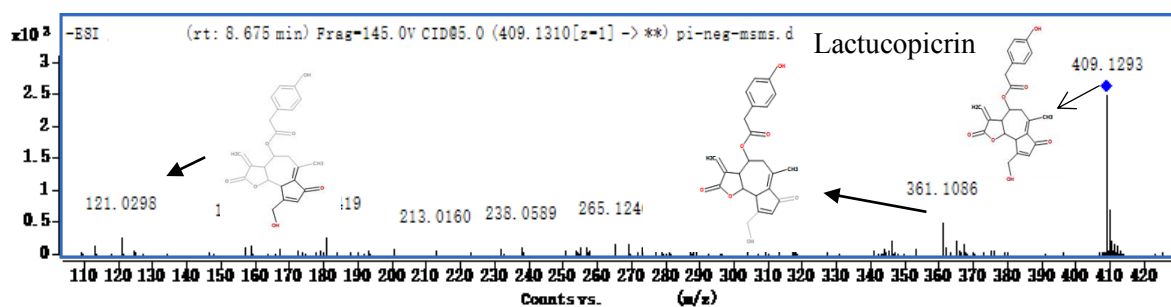

19

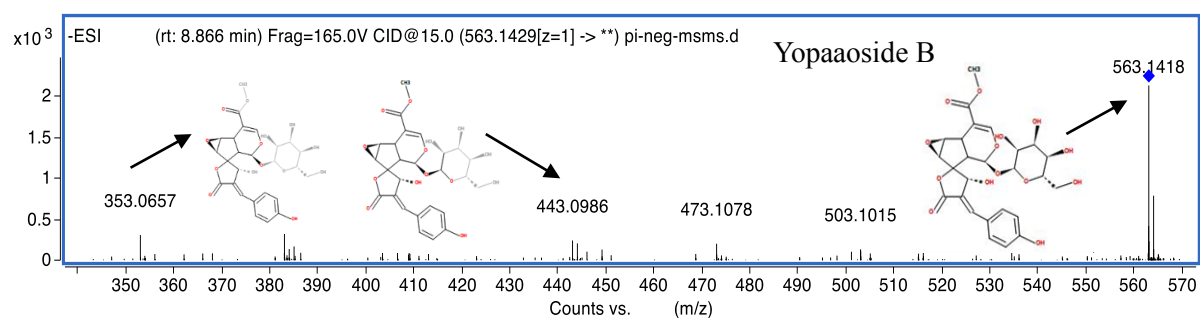

20

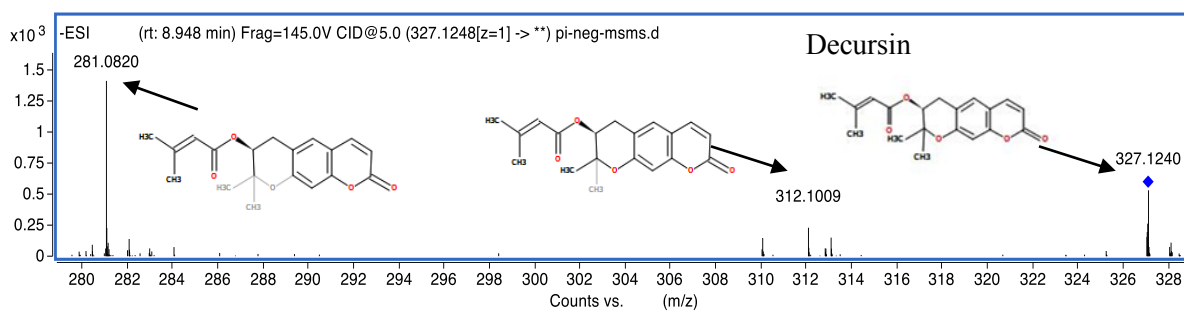

21

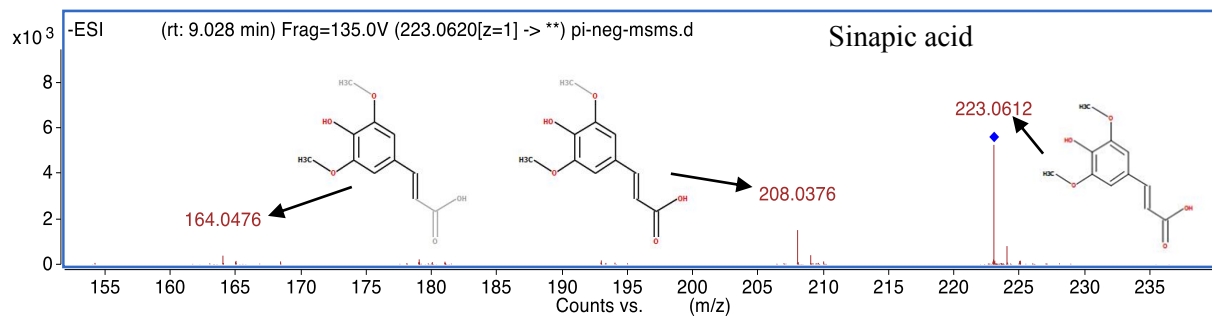

22

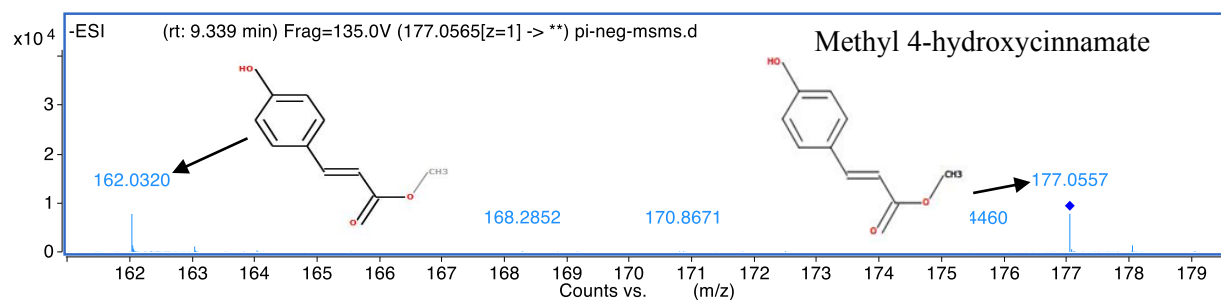

23

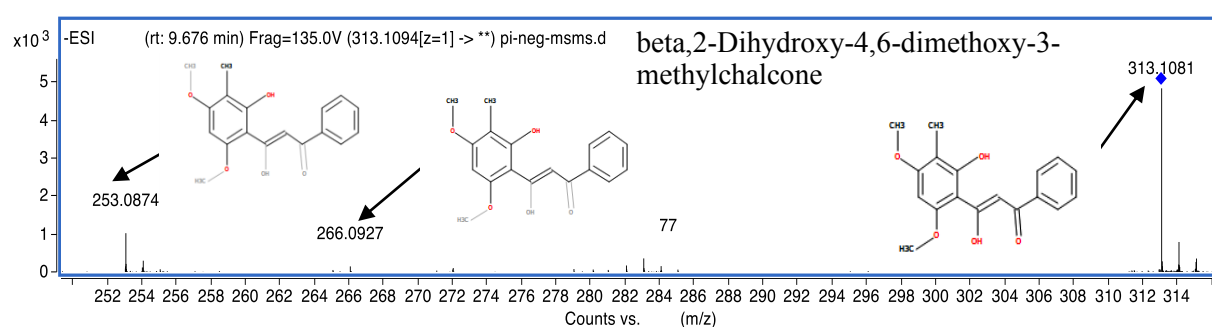

24

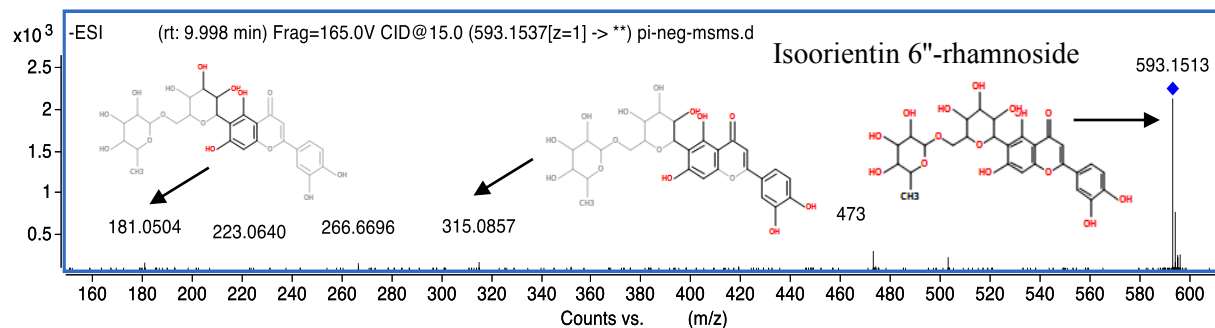

25

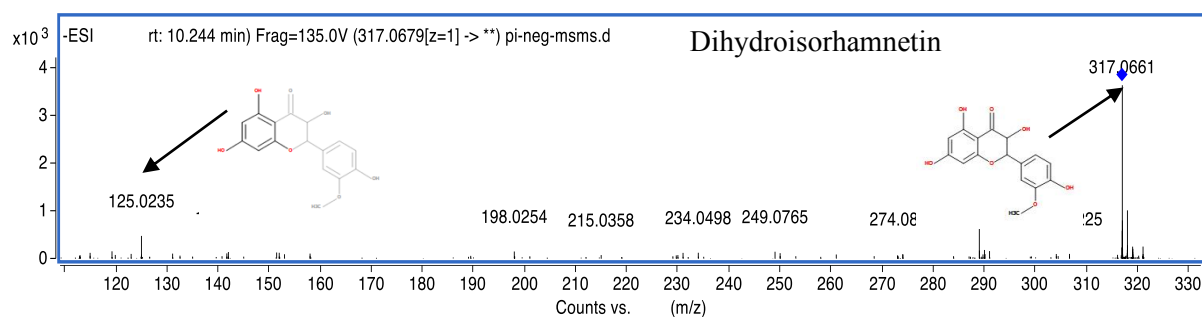

26

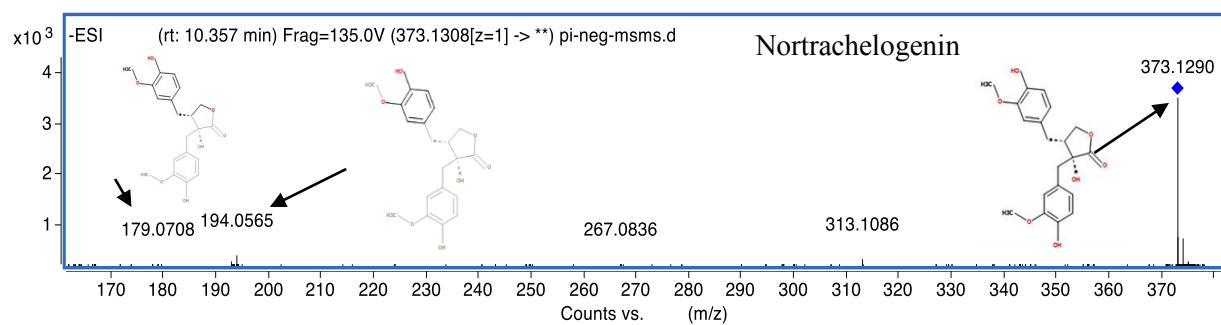

27

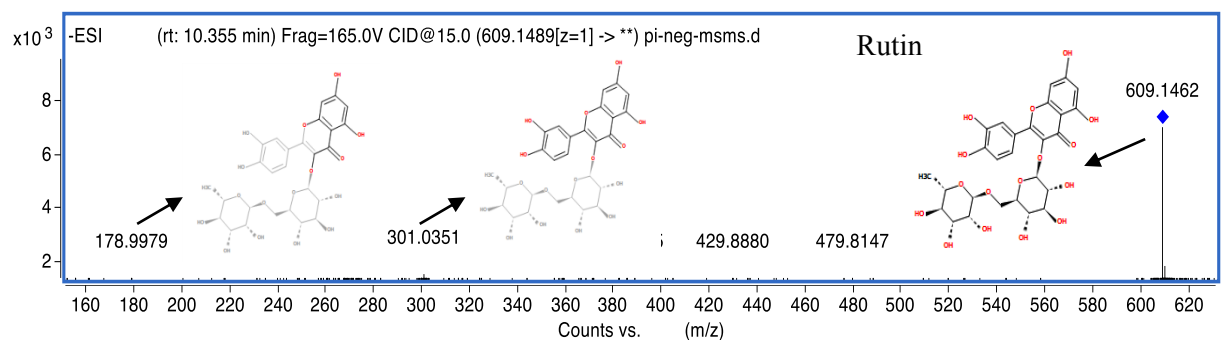

28

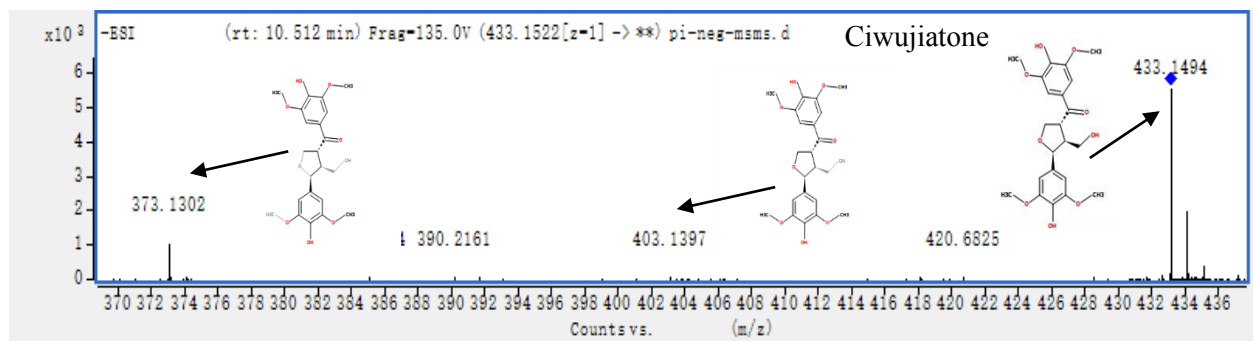

29

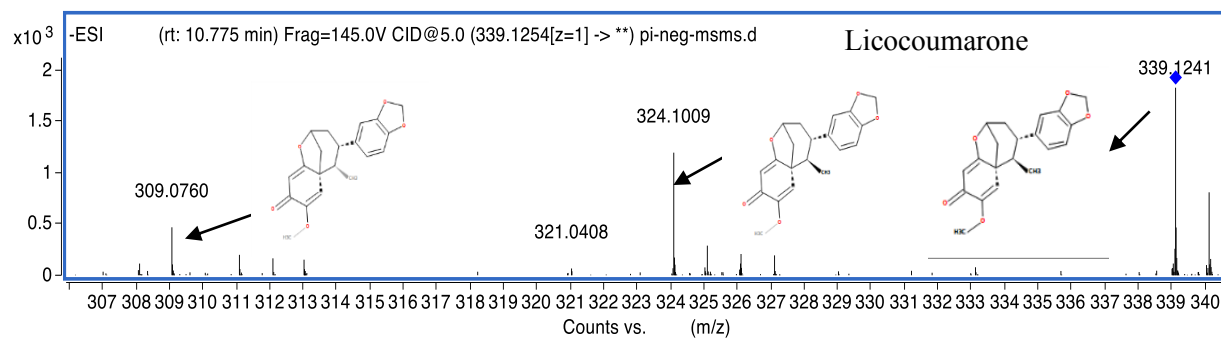

30

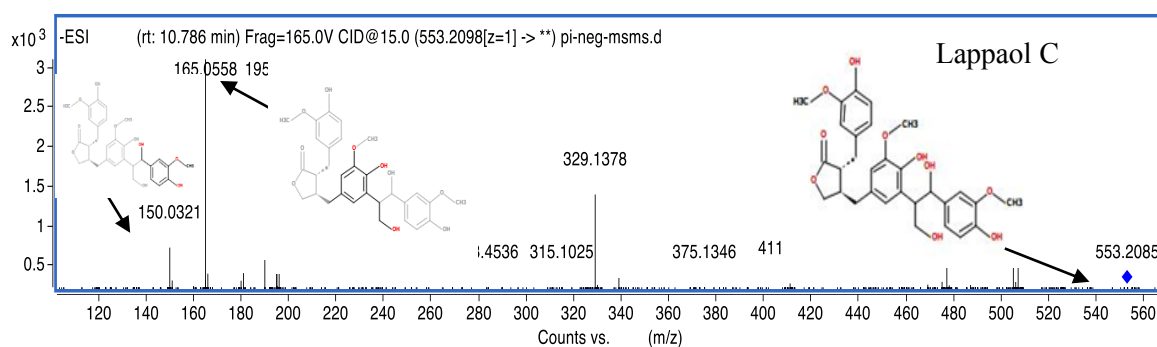

31

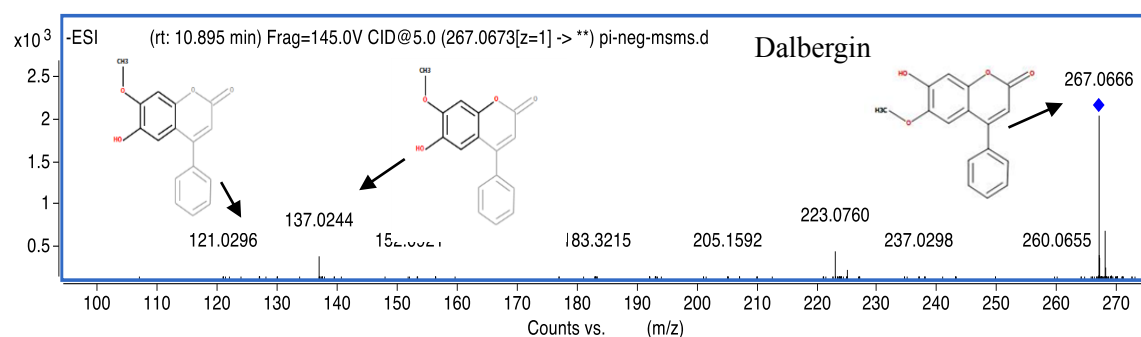

32

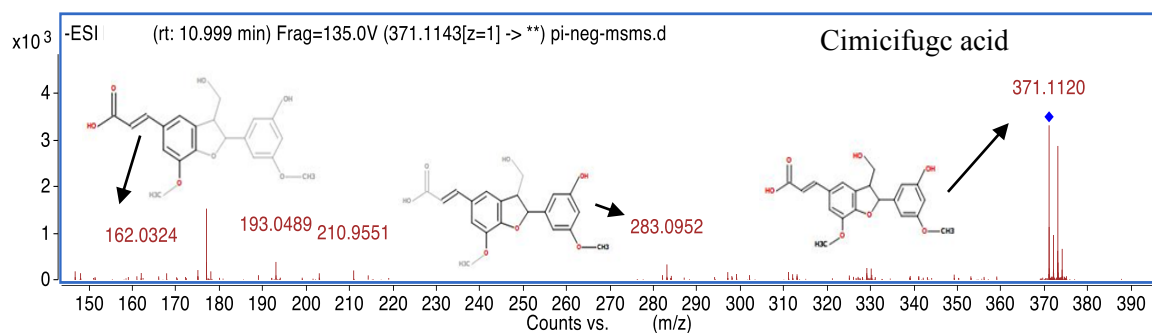

33

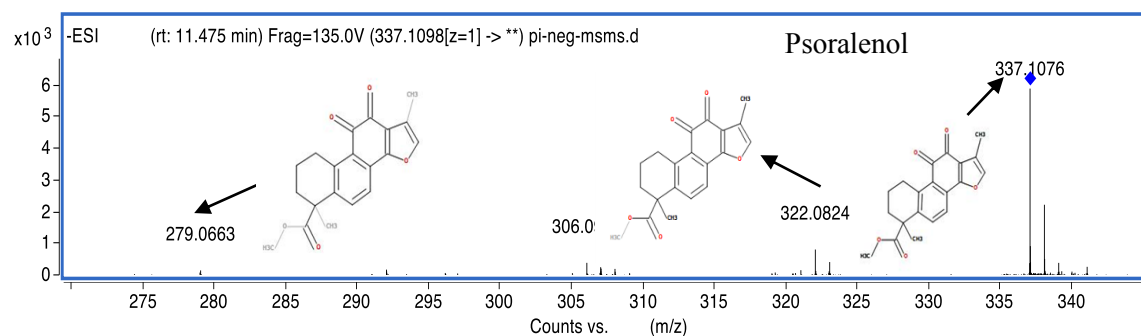

34

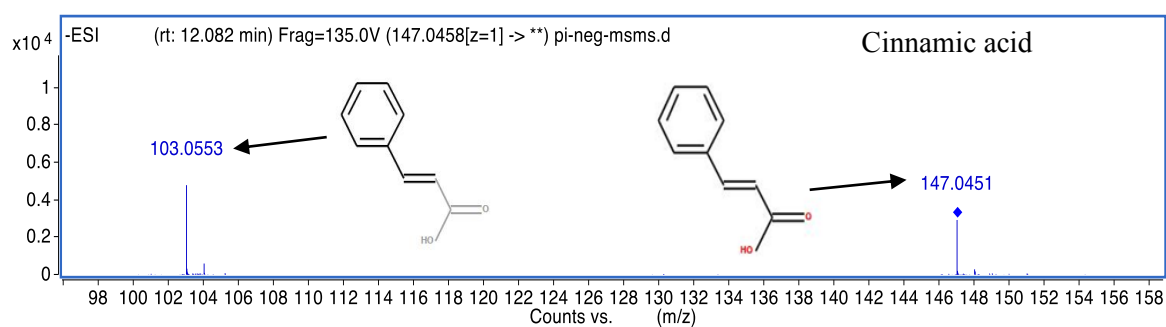

35

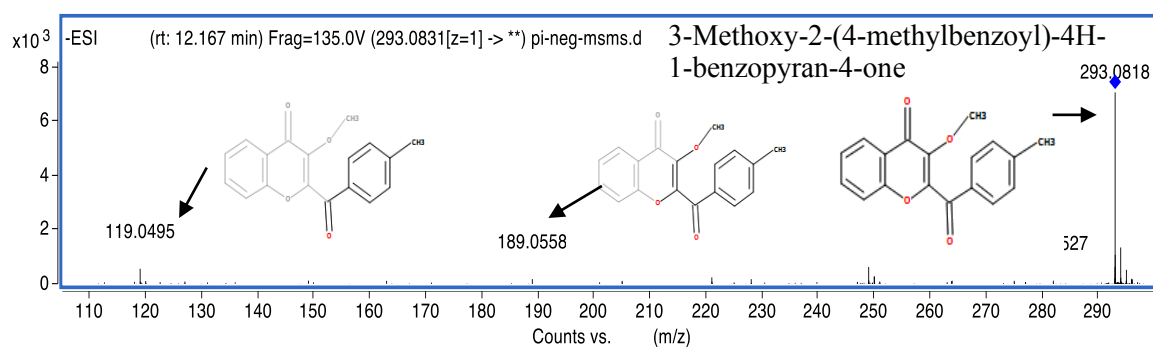

36

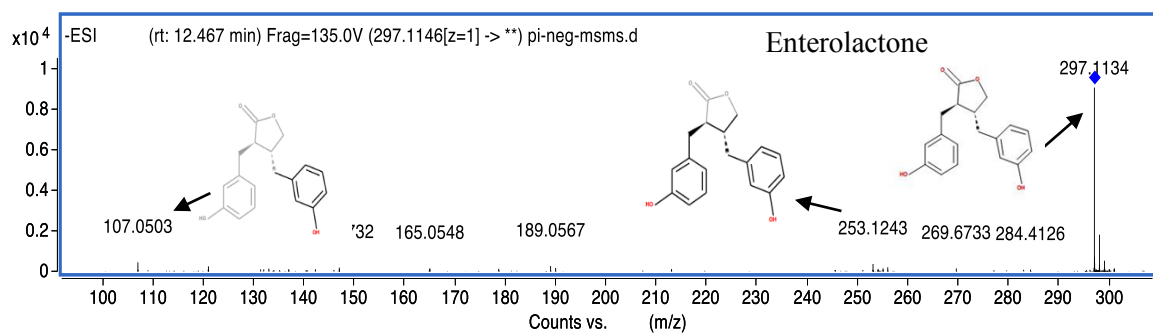

37

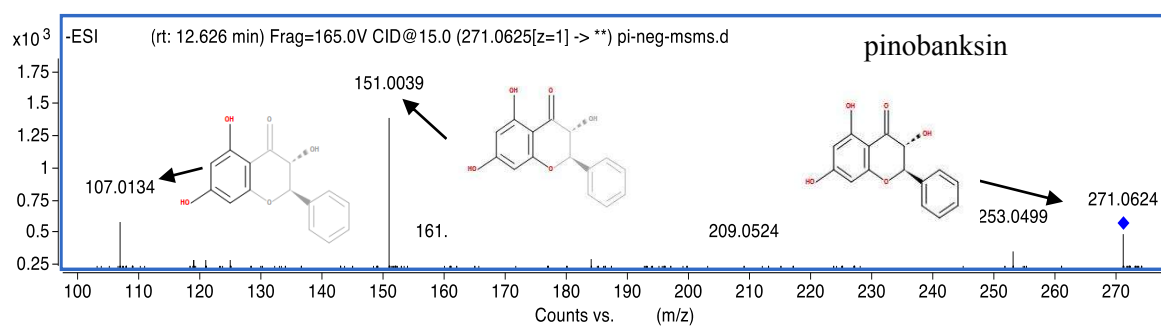

38

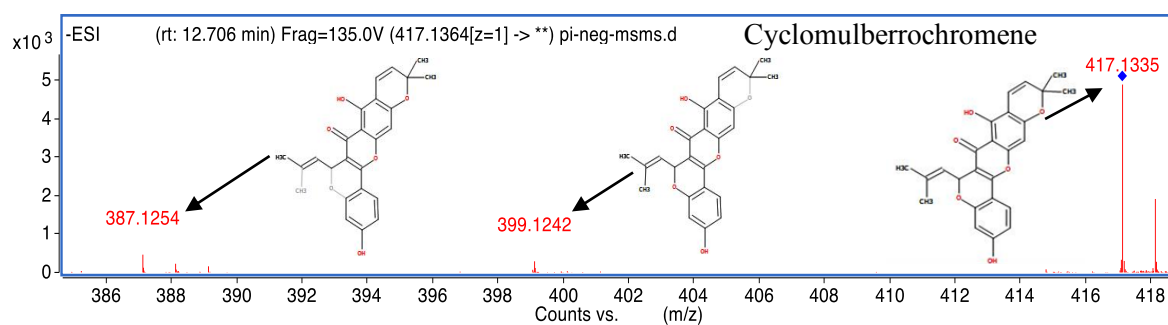

39

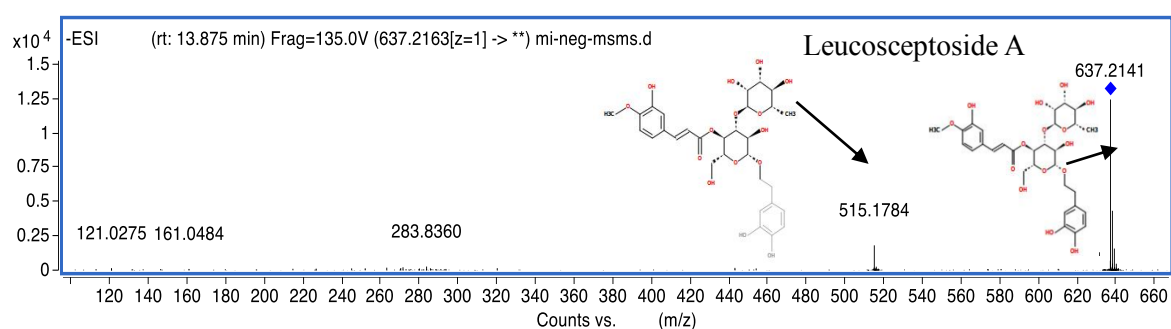

40

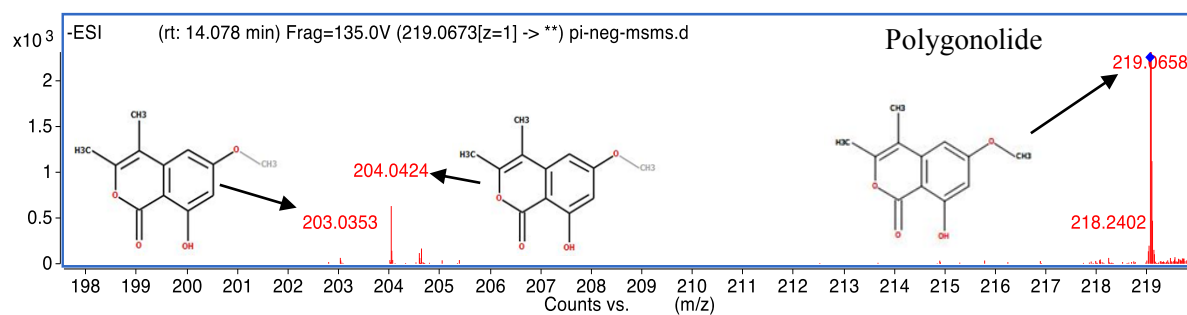

41

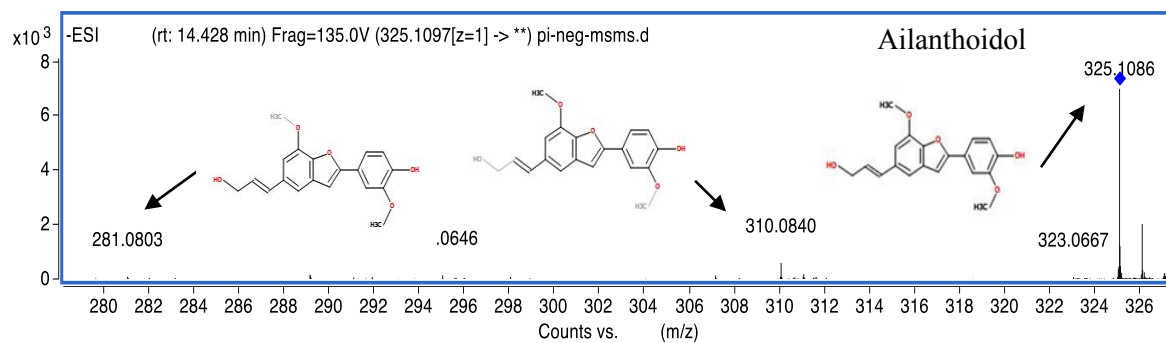

42

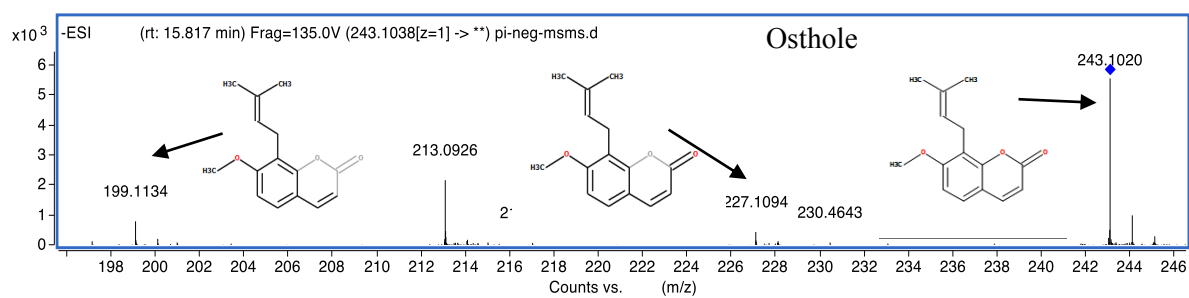

43

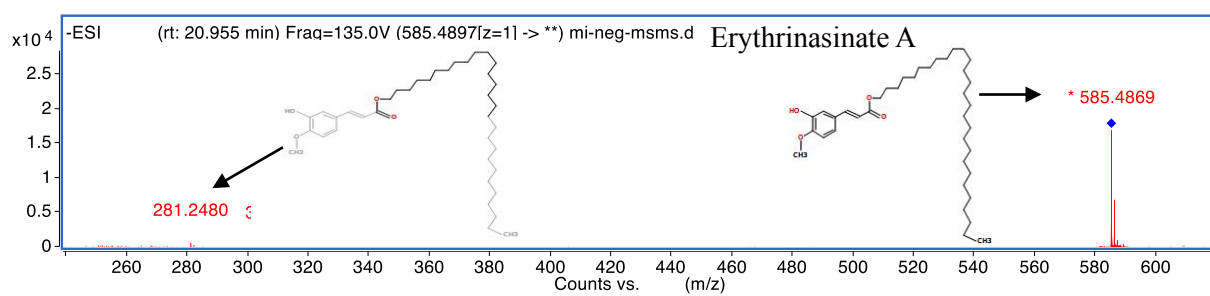

Supplement: Supplementary file 1 [file molecules-27-03767-s001.zip › molecules-1760775-supplementary.pdf]
